# CRISPR-Cas9-Mediated Mutagenesis of the Rubisco Small Subunit Family in *Nicotiana tabacum*

**DOI:** 10.3389/fgeed.2020.605614

**Published:** 2020-12-23

**Authors:** Sophie Donovan, Yuwei Mao, Douglas J. Orr, Elizabete Carmo-Silva, Alistair J. McCormick

**Affiliations:** ^1^SynthSys and Institute of Molecular Plant Sciences, School of Biological Sciences, University of Edinburgh, Edinburgh, United Kingdom; ^2^Lancaster Environment Centre, Lancaster University, Lancaster, United Kingdom

**Keywords:** chloroplast, *Chlamydomas reinhardtii*, photosynthesis, agroinfiltration, SpCas9, tobacco

## Abstract

Engineering the small subunit of the key CO_2_-fixing enzyme Rubisco (SSU, encoded by *rbcS*) in plants currently poses a significant challenge, as many plants have polyploid genomes and SSUs are encoded by large multigene families. Here, we used CRISPR-Cas9-mediated genome editing approach to simultaneously knock-out multiple *rbcS* homologs in the model tetraploid crop tobacco (*Nicotiana tabacum cv*. Petit Havana). The three *rbcS* homologs *rbcS_S1a, rbcS_S1b* and *rbcS_T1* account for at least 80% of total *rbcS* expression in tobacco. In this study, two multiplexing guide RNAs (gRNAs) were designed to target homologous regions in these three genes. We generated tobacco mutant lines with indel mutations in all three genes, including one line with a 670 bp deletion in *rbcS-T1*. The Rubisco content of three selected mutant lines in the T_1_ generation was reduced by *ca*. 93% and mutant plants accumulated only 10% of the total biomass of wild-type plants. As a second goal, we developed a proof-of-principle approach to simultaneously introduce a non-native *rbcS* gene while generating the triple SSU knockout by co-transformation into a wild-type tobacco background. Our results show that CRISPR-Cas9 is a viable tool for the targeted mutagenesis of *rbcS* families in polyploid species and will contribute to efforts aimed at improving photosynthetic efficiency through expression of superior non-native Rubisco enzymes in plants.

## Introduction

The assimilation of CO_2_ in photosynthetic organisms is primarily catalyzed by the bi-functional enzyme ribulose-1,5-biphosphate carboxylase/oxygenase (Rubisco). In plants, Rubisco has a relatively slow carboxylation rate ($k_{\text{cat}}^{\text{c}}$) and a competitive oxygenase activity that results in yield limitations, particularly in C_3_ plants, which include important crops such as *Oryza sativa* (rice) and *Triticum aestivum* (wheat). Variations in the catalytic properties of Rubisco between different species [e.g., carboxylation turnover rate ($k_{\text{cat}}^{\text{c}}$) and the specificity of Rubisco for CO_2_ vs. O_2_ (*S*_C/O_)] suggest that Rubisco could be engineered to improve the efficiency of CO_2_ assimilation in plants (Zhu et al., 2004; Galmés et al., 2014; Orr et al., 2016; Young et al., 2016; Martin-Avila et al., 2020).

Rubisco in plants (i.e., Form I Rubisco, L_8_S_8_) is composed of eight chloroplast-encoded (*rbcL* gene) large subunits (LSUs) that form the active sites, and eight small subunits (SSUs) that are nuclear-encoded by a family of *rbcS* genes (Spreitzer, 2003). Although structurally distant from the active site, SSUs are known to affect the catalytic properties of Rubisco (Genkov and Spreitzer, 2009; Ishikawa et al., 2011; Esquivel et al., 2013; Fukayama et al., 2019; Orr et al., 2020). Arabidopsis mutants that lack up to three out of four *rbcS* homologs have proven useful models for the expression of non-native SSUs to examine the effect of divergent sequences on Rubisco catalysis (Izumi et al., 2012; Atkinson et al., 2017; Khumsupan et al., 2020). However, replacing the native SSU family remains difficult in polyploid species (i.e., most crops), which can have up to 22 *rbcS* homologs (e.g., wheat) and tend to produce near-identical mature SSU peptides. Recently, a family of phylogenetically distinct *rbcS* homologs were identified in *Nicotiana tabacum* (tobacco), rice, and several other species that produce Rubisco with altered catalytic properties, including an increased $k_{\text{cat}}^{\text{c}}$ and decreased *S*_C/O_ (Morita et al., 2014, 2016; Laterre et al., 2017; Pottier et al., 2018). Although these *rbcS* homologs are typically expressed in non-photosynthetic tissues, overexpression could lead to changes in the catalytic properties of the Rubisco pool in leaves, provided that the remaining *rbcS* family members are sufficiently suppressed (Morita et al., 2016).

Tobacco and rice plants with reduced Rubisco content through antisense suppression of *rbcS* have offered an insight into the extent of Rubisco limitation on photosynthesis and growth and in response to different light intensities, nitrogen availability, and temperatures (Stitt and Schulze, 1994; Makino et al., 1997, 2000). For example, *rbcS* antisense tobacco mutants have shown that Rubisco content could be decreased to 40% of wild-type levels before impairment of growth and photosynthesis under controlled growth conditions (*ca*. 300 μmol photons m^−2^ s^−1^) (Quick et al., 1991a; Stitt et al., 1991). Although antisense studies have greatly advanced our understanding of Rubisco limitation in plants, the effectiveness of supression varies between plants, tissues, and developmental stages, and a loss of supression can occur in later generations (Quick et al., 1991b; Mitchell et al., 2004). As a result, the Rubisco content of each plant needs to be determined in every experiment, which affects extending this approach to engineer additional crop species and limits the potential to test large suites of candidate SSUs. Therefore, there is a need to generate lines with a stable suppression of endogenous SSUs to provide a platform to test SSU engineering approaches in crops.

Rubisco also requires several chaperone proteins for assembly, with significant progress made in recent years in establishing the assembly process in plants. For example, identifying and characterizing the roles of chaperone proteins has improved the efficiencies of producing chimeric Rubisco enzymes in tobacco and allowed the assembly of Rubisco from *Arabidopsis thaliana* (Arabidopsis) in *Escherichia coli* (Whitney et al., 2015; Aigner et al., 2017). Furthermore, enhancing plant productivity and robustness by increasing Rubisco abundance is now achievable in *Zea mays* (maize) through nuclear overexpression of native LSU, SSU and the RAF1 chaperone (Salesse-Smith et al., 2018, 2020) and in *O. sativa* (rice) through co-expressing an additional native SSU (Yoon et al., 2020). Recent work by Martin-Avila et al. (2020) described a next-generation tobacco mutant line (tobRrΔs) in which native Rubisco production was substituted with Rubisco from *Rhodospirillum rubrum* and native *rbcS* gene expression was blocked. Although the tobRrΔs mutant is an exciting screening platform for non-native Rubisco variants, routine expression of non-native Rubisco variants in wild-type crop plant backgrounds remains a significant challenge.

This goal is now feasible owing to the development of RNA-guided endonucleases (RGENs), such as CRISPR-Cas9, which facilitate the editing of multiple genes simultaneously in polyploid species (Morineau et al., 2017; Wolabu et al., 2020). Several toolkits have been developed for assembling plasmid vectors carrying multiple gRNA expression cassettes to target different genes (Xing et al., 2014; Lowder et al., 2015; Ma et al., 2015). Alternatively, gene families that share high nucleotide identity can be edited using one or more “promiscuous” gRNAs that target homologous regions (Endo et al., 2015). This approach was recently used to successfully induce frameshift mutations in *rbcS* genes in diploid rice (Matsumura et al., 2020). Here, we designed a CRISPR-Cas9 approach targeting the three predominant *rbcS* homologs in tobacco to explore the potential application of RGEN-mediated multigene editing of *rbcS* genes in a large, allotetraploid crop genome. The tobacco *rbcS* family comprises at least 13 homologs, and the three genes *rbcS-T1, rbcS-S1a*, and *rbcS-S1b* are reported to account for over 80% of total *rbcS* transcripts (Lin et al., 2020). We targeted these three genes and generated a tobacco triple SSU knockout mutant with reduced Rubisco content as a platform for heterologous SSU expression studies. We then tested a co-transformation strategy to simultaneously introduce a non-native *rbcS* gene while generating the triple SSU knockout, which reduces the need for multiple rounds of transformation and screening, and could benefit similar approaches in crop species that take longer to transform.

## Materials and Methods

### Plant Materials and Growth Conditions

Seeds of wild-type tobacco (*N. tabacum* cv. Petite Havana) and transgenic lines generated in this study were sown on a compost and sand mix (F2+ S; Levington, UK). Seeds were germinated in a controlled environment growth chamber (AR-36L3; Percival Scientific, USA) at 25°C and 60–70% relative humidity in a 16-h photoperiod with cool white fluorescent bulbs (170 μmol photons m^−2^ s^−1^). Fourteen-day-old seedlings were transplanted to pots (3L capacity) and maintained in a greenhouse (20–21°C day; 18°C night) in a 15-h photoperiod under natural light supplemented with 300 μmol photons m^−2^ s^−1^ of light. Plant positions were rotated every 2 days to allow consistent access to light and supplemented weekly with Hoagland solution (Hoagland and Snyder, 1933).

### gRNA Design

Two gRNAs with target sites common to exons 1 (gRNA1) and 4 (gRNA4) in *rbcS-S1a* (KM025316.1), *rbcS-S1b* (KM025317.1) and *rbcS-T1* were identified by the Cas-Designer tool (www.rgenome.net/cas-designer) as potential gRNA sites for editing by Cas9 from *Streptococcus pyogenes* (*spCas9*) using a dual gRNA approach (Bae et al., 2014; Park et al., 2015). We checked for potential off-target sites in the tobacco genome using the Cas-OFFinder tool (www.rgenome.net) and confirmed that the two gRNA sequences had no complementarity to any of the other ten *rbcS* homologs (Table 1) (Bae et al., 2014). A total of three potential off-target sites with two mismatches to the gRNA sequences were identified (Supplementary Table 1). The potential off-target sites were not evaluated further for off-target mutations as the mismatches were located in the 8–12 nt region proximal to the PAM site (Hahn and Nekrasov, 2019).

**Table 1:** Genomic locations of thirteen Rubisco small subunit genes in tobacco.

			***N. tabacum*** **TN90**^**b**^		***N. tabacum*** **v1.0**^**c**^	
**No**.	**Gene**	**Accession^**a**^**	**Scaffold**	**Location (bp)**	**Chr**.	**Location (bp)**
1	*rbcS-S1a*	KM025316.1	SS1336	810318–811169	Nt21	11725909–11726259
2	*rbcS-S1b*	KM025317.1	Maps to same region as *rbcS-S1a*	-	-	-
3	*rbcS-S2*	KM025319.1	SS4468	754873–755617	Nt03	46963643–46964387
4	*rbcS-S3*	KM025321.1	SS4468	554204–554937	Nt03	46873734–46874467
5	*rbcS-S4*	KM025323.1	SS4468	399989–400743	Nt03	46911953–46912707
6	*rbcS-S5*	KM025325.1	SS4468	456463–457081	Nt03	46771193–46772295
7	*rbcS-T1*	KM025327.1	SS2179	301404–302119	Nt14	90863242–90863574
8	*rbcS-T2*	KM025329.1	SS17012	102405–102957	Nt17	208193244–208193991
9	*rbcS-T3a*	KM025331.1	Maps to same region as *rbcS-T2*	-	Nt17	208121436–208122184
10	*rbcS-T3b*	KM025332.1	Maps to same region as *rbcS-T2*	-	Nt17	Maps to same region as *rbcS-T3a*
11	*rbcS-T4a*	KM025334.1	SS17012	88923–89705	Nt17	208180177–208180959
12	*rbcS-T4b*	KM025335.1	Maps to same region as *rbcS-T4a*			
13	*rbcS-T5*	KM025337.1	SS17012	138156–139280	Nt17	208193500–208193991

*The tobacco (N. tabacum) genome is allotetraploid with component diploid maternal and paternal genomes S and T, respectively, likely arising from hybridization between diploid N. sylvestris (S) and N. tomentosiformis (T) ancestors. Six Rubisco small subunit (rbcS) genes are on the S genome, and seven are on the T genome. Partial-coding sequences from (Gong et al., 2014^a^) were used to BLAST search two genome assemblies on the Sol Genomics database. The regions on the TN90 (Sierro et al., 2014^b^) and v.10 (Edwards et al., 2017^c^) genomes with the highest nucleotide identity to the target sequences were used to design gene-specific primers for this study*.

### Plasmid Design and Construction

All cloning reactions were performed in a 20 μL volume following the Golden Gate assembly protocol previously described (Engler et al., 2014). Plasmids pICSL90010 (Addgene #117520), pICSL90002 (Addgene #68261), pEPOR0SP0013 (Addgene #117521), and The MoClo Plant Parts Kit (Addgene kit # 1000000044) were gifts from Nicola Patron (Earlham Institute, UK) (Engler et al., 2014; Lawrenson et al., 2015; Raitskin et al., 2019). Full-length gRNA sequences were amplified from a template plasmid that contained the gRNA scaffold sequence (pICSL90010) and primers (IDT, Germany) that included a 19-nt protospacer region preceding the PAM site using Q5 High-Fidelity DNA Polymerase (M0491S; New England BioLabs, USA) as per the manufacturer's instructions (Supplementary Table 2). Each full-length gRNA sequence was assembled with the Arabidopsis *U6* gene promoter (pICSL90002) into a Level 1 entry vector. A coding sequence for *spCas9* optimized for expression in plants (pEPOR0SP0013) was assembled with the Arabidopsis *ubiquitin 10* promoter and 5′ untranslated region (UTR) (pICSL12015) and *heat shock protein 18.2* (HSP) terminator and 3′ UTR into a Level 1 entry vector (Nagaya et al., 2010). The four Level 1 assemblies carrying expression cassettes for spCas9, each of the two gRNAs, and kanamycin resistance were combined into a single Level 2 binary vector (pAGM4723) to produce the plasmid for plant transformation (pGRNA14) (Supplementary Figure 1). The vector used to express *rbcS2* from Chlamydomonas (*CrrbcS2*) was assembled by cloning the *CrrbcS2* coding sequence fused with the *rbcS1A* chloroplastic transit peptide sequence from Arabidopsis (Atkinson et al., 2017) into a Level 1 entry vector with the *S. lycopersicum rbcS2* (*SlrbcS2*) gene promoter and 5′ UTR (pICH71301) and HSP terminator into a Level 1 entry vector. The Level 1 *CrrbcS2* expression cassette was assembled with a hygromycin resistance cassette into a Level 2 binary vector for plant transformation (pRBCS-Cr) (Supplementary Figure 2).

### Tobacco Transformation

Vectors were transformed into *Agrobacterium tumefaciens* strain AGL1 by electroporation and colonies were verified by PCR and sequencing using insert-specific primers (Supplementary Table 2). For transient expression in tobacco leaves, a 15 mL culture was prepared, resuspended in 10 mM MgCl_2_ to an OD_600_ of 0.8. Diluted cultures were syringe-infiltrated into the youngest fully expanded leaves of four-week-old plants. Stable CRISPR-Cas9 lines were produced by germinating sterile wild-type seeds in Magenta GA-7 boxes (V8505; Sigma Aldrich, UK) on 0.8% (w/v) agar (pH 5.8) containing Murashige and Skoog (MS) medium (M5524; Sigma Aldrich) and 3% (w/v) sucrose. A 150 mL suspension of AGL1 containing vector pGRNA14 was prepared and resuspended in the same volume of 1x liquid MS. Leaves from 6-week-old plants were cut into 2 cm^2^ pieces, incubated for 30 min in the AGL1 suspension, and placed abaxial side up on MS medium containing 0.1 mg/L indole-3-butyric acid (IBA) (57310; Sigma Aldrich) and 1 mg/L 6- benzylaminopurine (B3408; Sigma Aldrich). After 2 days of co-cultivation with *Agrobacterium*, leaf discs were washed three times in liquid MS and cultured on selective media with 500 mg/L augmentin and 100 mg/L kanamycin to select for pGRNA14. Shoots were excised after 4–5 weeks and placed on MS medium with 100 mg/L kanamycin in Magenta GA-7 boxes for root induction. Following the appearance of roots, kanamycin-resistant plantlets were transferred to pots (9 cm diameter) and leaf tissue was harvested to screen for mutations in T_0_ lines by PCR (see section Mutation Screening). Seeds from T_0_ plants were germinated on soil and screened for mutations by PCR to obtain T_1_ plants for the growth analysis.

Stable CRISPR-Cas9 lines overexpressing *CrrbcS2* were generated as previously described except that explants were cultured on media that contained 100 mg/L kanamycin and 30 mg/L hygromycin to select for pGRNA14 and pRBCS-Cr, respectively. The T_1_ generation of plants was obtained by germinating seeds on MS that contained 30 mg/L hygromycin to select for pRBCS-Cr and screened for mutations by PCR.

### Mutation Screening

Genomic DNA was extracted as previously described (Khumsupan et al., 2020). Kanamycin-resistant plantlets were first screened by PCR using primers for *spCas9* to confirm the presence of the transgene. Gene-specific primers for the *rbcS* genes were designed based on the tobacco draft genomes and used to amplify *rbcS-T1* (rbcS-T1_F1 and rbcS-T1_R1) and *rbcS-S1a/b* (rbcS-S1_F1 and rbcS-S1_R1) (Sierro et al., 2014; Edwards et al., 2017). All primer sequences are given in Supplementary Table 2 (Gong et al., 2014). Sanger sequencing of PCR amplicons was performed by Edinburgh Genomics (Edinburgh, UK). Mutations were identified by pairwise sequence alignment with the respective wild-type genomic DNA sequences using EMBOSS Needle (EMBL-EBI, UK) (Madeira et al., 2019). Mutation frequencies were determined from the sequencing chromatograms using TIDE (http://tide.deskgen.com) (Brinkman et al., 2014).

### RNA Extraction and qRT-PCR

Total RNA was isolated from leaf tissue using an RNeasy Plant Mini Kit (#74904, QIAGEN) and treated with RNase free DNase I (#79254, QIAGEN) according to the manufacturer's protocol. For cDNA synthesis, 1 μg of RNA was reverse-transcribed in a 20 μL reaction according to the protocol for the GoScript Reverse Transcription System (A5003, Promega, USA). Quantitative reverse transcription PCR (qRT-PCR) reactions were prepared in a 10 μL volume that contained 4 μL cDNA (8 ng/μL), 1 μL of each primer (10 μM) and 5 μL of SYBR Mastermix (B0701, Eurogentec, Belgium) and performed on a LightCycler 480 (05015278001, Roche, Switzerland) with the following thermal cycling parameters: 95°C for 3 min, 40 cycles of 95°C for 10 s, 60°C for 20 s, 72°C for 30 s followed by a dissociation curve (66–95°C) at the end of each run. Relative expression of the target genes was calculated according to the 2^−ΔΔCt^ method using the tobacco *ribosomal protein L25* gene (GenBank: L18908) for normalization (Schmidt and Delaney, 2010). All primer sequences are given in Supplementary Table 3.

### Protein Extraction and Western Blotting

*Chlamydomonas reinhardtii* (Chlamydomonas) cultures were provided as a gift from Attila Molnar (University of Edinburgh, UK). A cell lysate was prepared from Chlamydomonas cells according to (Atkinson et al., 2019). Leaf samples (7.9 cm^2^) were harvested and immediately frozen and total protein was extracted in 25 mM Tris-HCl (pH 7.5), 1 mM EDTA, 10% (v/v) glycerol, 0.1% (v/v) TritonX-100, 150 mM NaCl, 1 mM DTT and cOmplete^TM^ EDTA-free protease inhibitor cocktail (COEDTAF, Roche). The sample was centrifuged at 5,000 g (4°C) for 5 min and a sub-sample of the supernatant (10 μL) was combined with Pierce 660 nm Protein Assay Reagent (22660, ThermoFisher, UK) to measure total soluble protein against BSA pre-diluted standards (23208, ThermoFisher). The remaining sample was mixed with 1% (w/v) LDS and 1 μL (per 100 μL) β-mercaptoethanol, and heated to 100°C for 1 min. Total soluble protein was separated by SDS-PAGE on 12% Bis-Tris gels (NP0342, Invitrogen, USA) and transferred to a PVDF membrane using iBlot2 gel transfer (IB21001, Invitrogen). Membranes were probed with rabbit serum raised against wheat Rubisco (Howe et al., 1982) at a 1:10000 dilution, RbcS2 from Chlamydomonas (CrRbcS2) (raised to the C-terminal region of the SSU (KSARDWQPANKRSV) by Eurogentec, Southampton, UK) at 1:1000 dilution, histone H3 (ab18521, Abcam, UK) at a 1:10000 dilution, or actin (60008-1-1G, Proteintech, USA) at a 1:1000 dilution. A 1:10000 dilution of IRDye 800CW goat anti-rabbit IgG (LI-COR, USA) was used to visualize bands on an Odyssey Clx Imager (LI-COR) that were quantified with Image Studio Lite software (v. 5.2.5, LI-COR).

### Rubisco Content

Leaf samples (5.9 cm^2^) were collected from the youngest fully expanded leaves of 4-week-old plants, frozen on liquid nitrogen and stored at −80 °C before extraction. Samples were ground rapidly in an ice-cold mortar and pestle in 250 μL of extraction buffer (50 mM Bicine-NaOH pH 8.2, 20 mM MgCl2, 1 mM EDTA, 2 mM benzamidine, 5 mM ε-aminocaproic acid, 50 mM β-mercaptoethanol, 10 mM dithiothreitol, 1% (v/v) protease inhibitor cocktail (Sigma-Aldrich, Mo, USA), and 1 mM phenylmethylsulphonyl fluoride) for *ca*. 1 min followed by 1 min centrifugation (14,700 g at 4°C). The supernatant (100 μL) was then mixed with 100 μL of carboxyarabinitol-1,5-bisphosphate (CABP) binding buffer which contained 100 mM Bicine-NaOH (pH 8.2), 20 mM MgCl_2_, 20 mM NaHCO_3_, 1.2 mM (37 kBq μmol^−1^) [^14^C]CABP, incubated at RT for 25 min, and Rubisco content determined via [^14^C]CABP binding (Sharwood et al., 2016). Bradford assay was used to determine total soluble protein in the same supernatant as prepared for Rubisco content analysis (Bradford, 1976).

### Photosynthesis Measurements

The response of photosynthesis (*A*) to different levels of photosynthetic active radiation (PAR) (1,800, 1,500, 1,000, 500, 200, 100, 50, and 20 μmol photons m^−2^ s^−1^) was measured at 400 μmol CO_2_ mol^−1^ using a LI-COR 6400-XT portable gas exchange system (LI-COR) on 41–44 day-old plants. The response of *A* to the intercellular CO_2_ concentration (*C*_i_) was measured under a saturating light intensity (1,500 μmol photons m^−2^ s^−1^). The first measurement of each *A*/*C*_i_ curve was taken at an external CO_2_ concentration (*C*_a_) of 400 μmol mol^−1^ and then decreased to 50 μmol mol^−1^ in increments of 50 μmol mol^−1^. The upper part of the *A*/*C*_i_ response was measured from 500 to 2,000 μmol mol^−1^ in increments of 200 μmol mol^−1^. All measurements were taken with leaf temperature held at 25 °C and under a relative humidity of 60–70%. The maximum rate of photosynthesis at ambient levels of CO_2_ (*A*_sat_) was estimated from the *A*/PAR response as described in Monteith (1991). The maximum carboxylation rate of Rubisco (*V*_cmax_) was estimated by fitting *A*/*C*_i_ data to a C_3_ photosynthesis model (Ethier and Livingston, 2004). Dark-adapted leaves were used to determine the maximum quantum yield of photosystem II (*F*_v_/*F*_m_) using a Handy PEA chlorophyll fluorimeter (Hansatech Instruments, UK). *F*_v_/*F*_m_ measurements were taken on the final day of growth experiments prior to harvesting.

### Chlorophyll Content

Chlorophyll was extracted on a leaf area basis (58.9 mm^2^) as described in Khumsupan et al. (2020) and quantified according to Porra et al. (1989).

### Growth Measurements

Plants in the growth experiments were harvested at 45-days old for growth measurements and the leaves and stems were separated immediately and weighed to determine the fresh weight. Images of separated stems and leaves were analyzed to determine total leaf area and stem height using iDIEL Plant software and ImageJ, respectively (Schneider et al., 2012; Dobrescu et al., 2017). Samples were then dried in an oven (80°C for 7 days) and weighed to determine dry weight.

### Statistical Analysis

All statistical analyses were performed using GraphPad Prism 8 software (GraphPad Software, USA). Significant differences between two groups were identified using Student's *t*-test (*P* < 0.05) and more than two groups were evaluated using a one-way ANOVA followed by Tukey's honestly significant difference (HSD) test (*P* < 0.05).

## Results

### CRISPR-Cas9 Was Highly Efficient in Tobacco Transient Expression Assays

**Figure 1:**
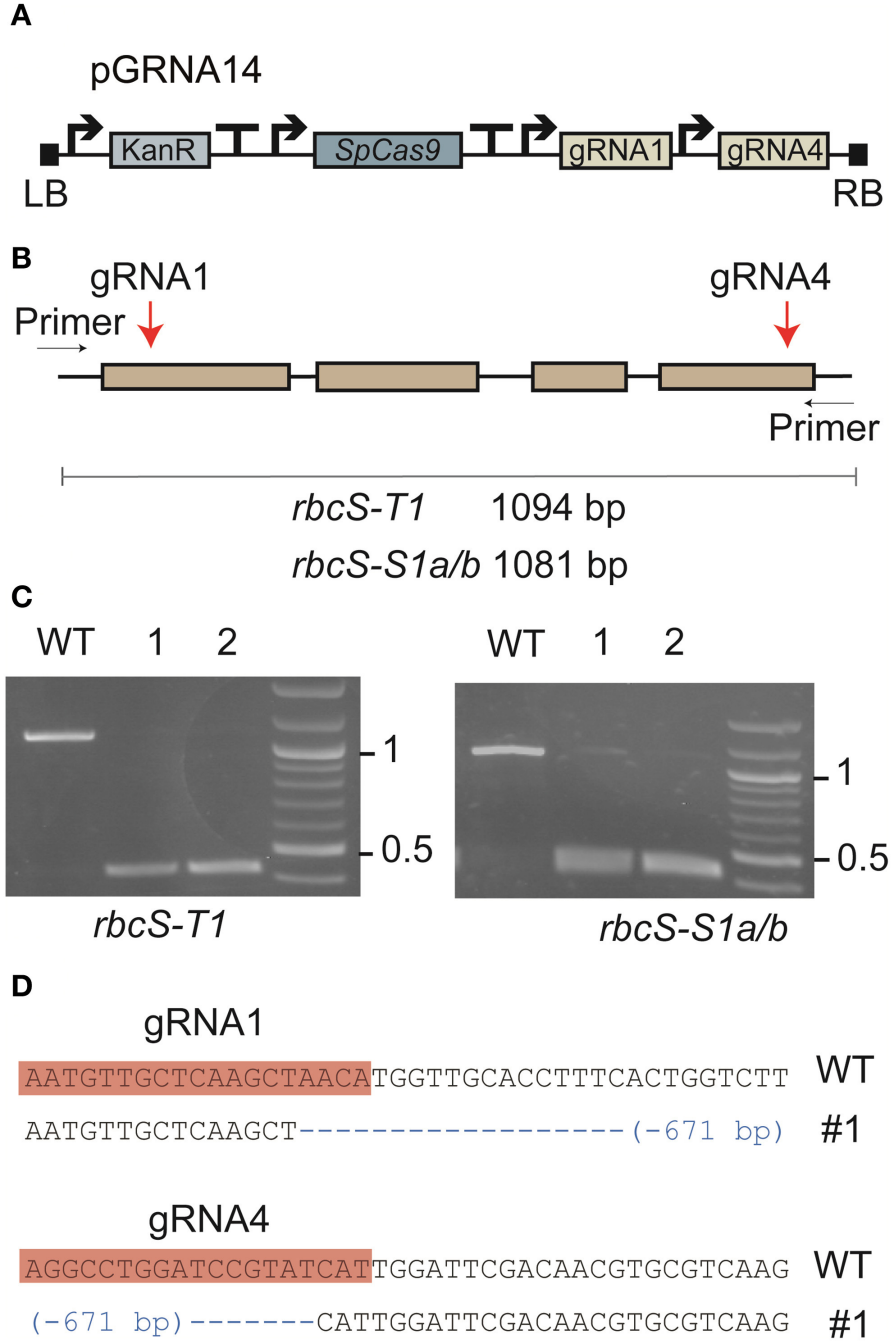
Expression of a Cas9-gRNA vector targeting three Rubisco small subunit genes in tobacco. **(A)** Cas9-gRNA expression construct with *Cas9* from *Streptococcus pyogenes* (SpCas9) and two gRNA sequences (gRNA1 and gRNA4) (see Supplementary Figure 1 for details of vector pGRNA14). **(B)** The target sites of gRNA1 and gRNA4 were located on the first and fourth exons, respectively, in *rbcS-T1, rbcS-S1a*, and *rbcS-S1b*. **(C)** PCR screen of leaf tissue with pGRNA14 transiently expressed showed deletion events (i.e., bands at <500 bp) in *rbcS-T1* (left) and *rbcS-S1a/b* (right). **(D)** Example of a pairwise sequence comparison of the deletion band from sample 1 (#1) for *rbcS-T1* showed a 671 bp deletion between the gRNA1 and gRNA4 target sites compared to wild-type (WT) *rbcS-T1*.

Two gRNAs (gRNA1 and gRNA4) were designed to target DNA sequence regions found only in the three most highly expressed *rbcS* homologs in tobacco (*rbcS-T1, rbcS-S1a*, and *rbcS-S1b*) (Figures 1A,B) (Lin et al., 2020). Gene-specific primers for screening edits in *rbcS-S1a* or *rbcS-S1b* were not available when the gRNA sites were initially designed, as the only available reference genes were partially sequenced, 98.5% identical, and did not map to unique regions on the tobacco KN90 genome (Sierro et al., 2014) (Table 1, Supplementary Figure 3). To try to overcome this limitation, we used a paired gRNA approach to generate a large deletion in each *rbcS* gene that could be screened using primers common to both *rbcS-S1a* and *rbcS-S1b*.

Vector pGRNA14 (carrying expression cassettes for gRNA1, gRNA4, and *SpCas9*) was transiently expressed in tobacco leaves by agroinfiltration to test the efficiency of the gRNAs. Subsequent amplification of DNA from agro-infiltrated leaves showed that the expected amplicons for wild-type *rbcS-T1* and *rbcS-S1a/b* were absent or barely detectable compared to the wild-type control (Figure 1C). Instead, smaller amplicons were observed that were consistent with a large deletion event between the gRNA 1 and gRNA four sites. The substantial reduction in intensity of the wild-type amplicons for *rbcS-S1a/b* suggested that both *rbcS-S1a* and *rbcS-S1b* had been edited. Sequencing of *rbcS-S1a/b* amplicons was not performed because sequence-specific primers to distinguish the two orthologs were unavailable. However, sequencing of the smaller sized band for *rbcS-T1* confirmed that a 671 bp deletion had occurred between the gRNA1 and gRNA4 sites (Figure 1D). Therefore, transient expression assays clearly showed that gRNA1 and gRNA4 were functional and appeared highly efficient in tobacco.

### Stable and Chimeric Mutations Were Identified in the T_0_ Generation

**Figure 2:**
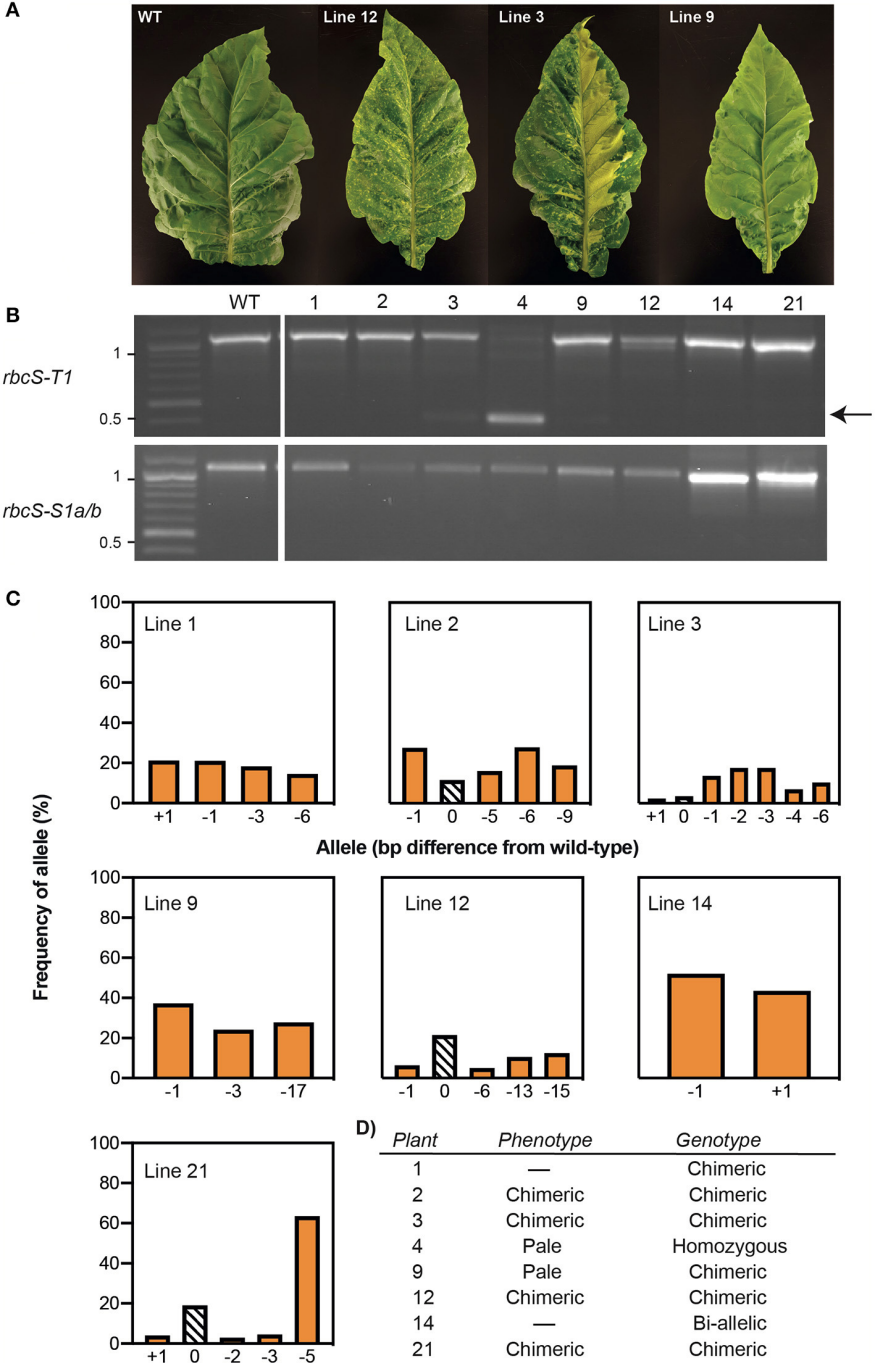
Phenotypes and genotypes of tobacco plants transformed with Cas9-gRNA vector pGRNA14. **(A)** T_0_ plants had paler leaves than wild-type (WT) (e.g., line 9) or chimeric leaf phenotypes (e.g., lines 3 and 12). **(B)** A PCR screen of *rbcS-T1* and *rbcS-S1a/b* in eight T_0_ lines identified a deletion between gRNA1 and gRNA4 in *rbcS-T1* in line 4 (arrow) and wild-type-sized amplicons for the remaining plants. **(C)** TIDE analysis of the sequencing chromatograms for wild-type-sized amplicons of *rbcS-T1* showed the frequency of wild-type (0 bp) or mutant [insertion (+N bp) or deletion –N bp)] alleles. **(D)** Summary of phenotypes and *rbcS-T1* genotypes of T_0_ plants.

Tobacco leaf disks were transformed with vector pGRNA14 and cultured on selective media to obtain kanamycin-resistant plantlets. Eight T_0_ plants with varying leaf phenotypes were transferred to soil and confirmed to contain the *SpCas9* transgene by PCR using primers specific for the *SpCas9* expression cassette. Four of these had visibly smaller and paler leaves than the non-transformed tissue culture control (i.e., wild-type), which is typical of Rubisco-deficient mutants (Khumsupan et al., 2020; Martin-Avila et al., 2020) (Figure 2A). The remaining four plants had a mixed pale and wild-type leaf phenotype indicative of chimeric mutations. All eight plants were screened for mutations in *rbcS-T1* and *rbcS-S1a/b* by PCR using the same primers as used for the transient expression assays (Figure 2B). In contrast to the results for the latter, only a single plant (line 4) showed a deletion band for *rbcS-T1*, while all other amplifications were similar in size to wild-type amplicons. Sequencing of the *rbcS-T1* deletion band from line 4 confirmed a 670 bp deletion between the two gRNA sites (Supplementary Figure 4). Therefore, line 4 was considered homozygous for the 670 bp deletion because only a single allele was identified by PCR and sequencing.

The wild-type sized amplicons for *rbcS-T1* from the remaining seven plants were sequenced to assess if small mutations were present. Pairwise-sequence alignments between the *rbcS-T1* amplicons from the transgenic plants and wild-type suggested that more than one *rbcS-T1* allele was present in each plant. The TIDE tool was used to identify and determine the frequency of different mutations in samples that likely had more than one allele by analyzing the sequencing chromatograms from mutant and wild-type plants (Figure 2C) (Brinkman et al., 2014). Plants with one mutated allele in addition to the wild-type allele were considered heterozygous, plants with two mutated alleles were considered bi-allelic, and those with more than two alleles were considered chimeric (Figure 2D). The TIDE analysis identified mutations at the gRNA4 target site that ranged in size from +1 bp to −17 bp between the seven lines. No mutations were observed at the gRNA1 target site. Line 14 appeared to have a bi-allelic mutation in *rbcS-T1* (i.e., a 1 bp deletion and 1 bp insertion) and had pale leaves similar to line 4. The remaining six plants had three or more alleles. The wild-type allele was identified in lines 2, 3, 12, and 21, which was consistent with the chimeric leaf phenotype seen for these plants. In contrast, only mutant alleles were identified in lines 1 and 9, both of which had homogenous pale leaves.

Similar to the transient expression assays, it was not possible to inspect for mutations in *rbcS-S1a* and *rbcS-S1b* by sequencing. T_0_ plant lines 1 and 14 did not establish following transfer to soil, but all other lines were progressed to the T_1_ generation based on the observed phenotypes and evidence of mutations in *rbcS-T1*.

### Transgene-Free Mutants Were Identified in the T_1_ Generation

To identify heritable mutations in *rbcS-T1* in the T_1_ generation, we first screened for the absence of the *SpCas9* transgene in the progeny of lines 2, 3, 4, 9, 12, and 21 (Table 2). Transgene-free T_1_ plants accounted for 15% of line 2 (2/13), 79% of line 3 (15/19), 32% of line 4 (5/19), 56% of line 9 (10/18), 11% of line 12 (2/19). No transgene-free plants were identified in line 21. A range of phenotypes were observed among transgenic and transgene-free T_1_ plants in the different lines. All plants for line 21 had a chimeric phenotype. Plants from lines 2 and 3 that retained *SpCas9* had pale leaves but the transgene-free progeny of these lines appeared similar to wild-type. However, all plants from lines 4, 9, and 12 had a pale leaf phenotype regardless of the absence or presence of *SpCas9*.

**Table 2:** Inheritance of CRISPR-Cas9 mutations in the Rubisco small subunit *rbcS-T1*.

**Line**	**T_**0**_ generation**	**T_**1**_ generation**	**Alleles (T_**1**_)**
2	Chimeric	3bi;5chi	d1/d5 (2)
			d9/d6 (1)
3	Chimeric	1bi;4het;10chi	d2/i1 (1)
			d2/WT (3)
			i1/WT (1)
4	Homozygous	16hom	d670 (16)
9	Chimeric	2hom;2bi;4chi	d1 (2)
			d17/d3 (1)
			d1/**d6** (1)
12	Chimeric	6bi; 6chi	d2/**d7** (2)
			d1/d6 (1)
			d9/**d4** (3)
21	Chimeric	n/a	n/a

*The number of T_1_ progeny with homozygous (hom), heterozygous (het), bi-allelic (bi) or chimeric (chi) mutations is shown. Non-chimeric alleles in the T_1_ generation are described as deletions (d) or insertions (i) followed by the number of base-pairs compared to wild-type (WT). Alleles that were not identified in the T_0_ progenitor are shown in bold lettering. The number of progeny with a single genotype is subsequently shown in brackets*.

Owing to the consistent pale leaf phenotype, we screened for mutations in *rbcS-T1* in T_1_ plants of lines 4, 9, and 12 (Table 2). All line four plants appeared homozygous for the 670 bp deletion allele of *rbcS-*T1. Consistent with the T_0_ generation, only wild-type-sized amplicons were observed for lines 9 and 12. Sequencing the amplicons of four line nine plants showed a variety of inherited mutations around the gRNA4 target site: two plants had a 1 bp homozygous deletion, two plants had different bi-allelic mutation (i.e., a 17 bp deletion and 3 bp deletion, and a 1 bp deletion and 6 bp deletion, respectively). Amplicons of the two line 12 plants also revealed bi-allelic mutations: one plant contained a 1 bp deletion and 6 bp deletion, and the other had a 9 bp deletion and 4 bp deletion.

**Figure 3:**
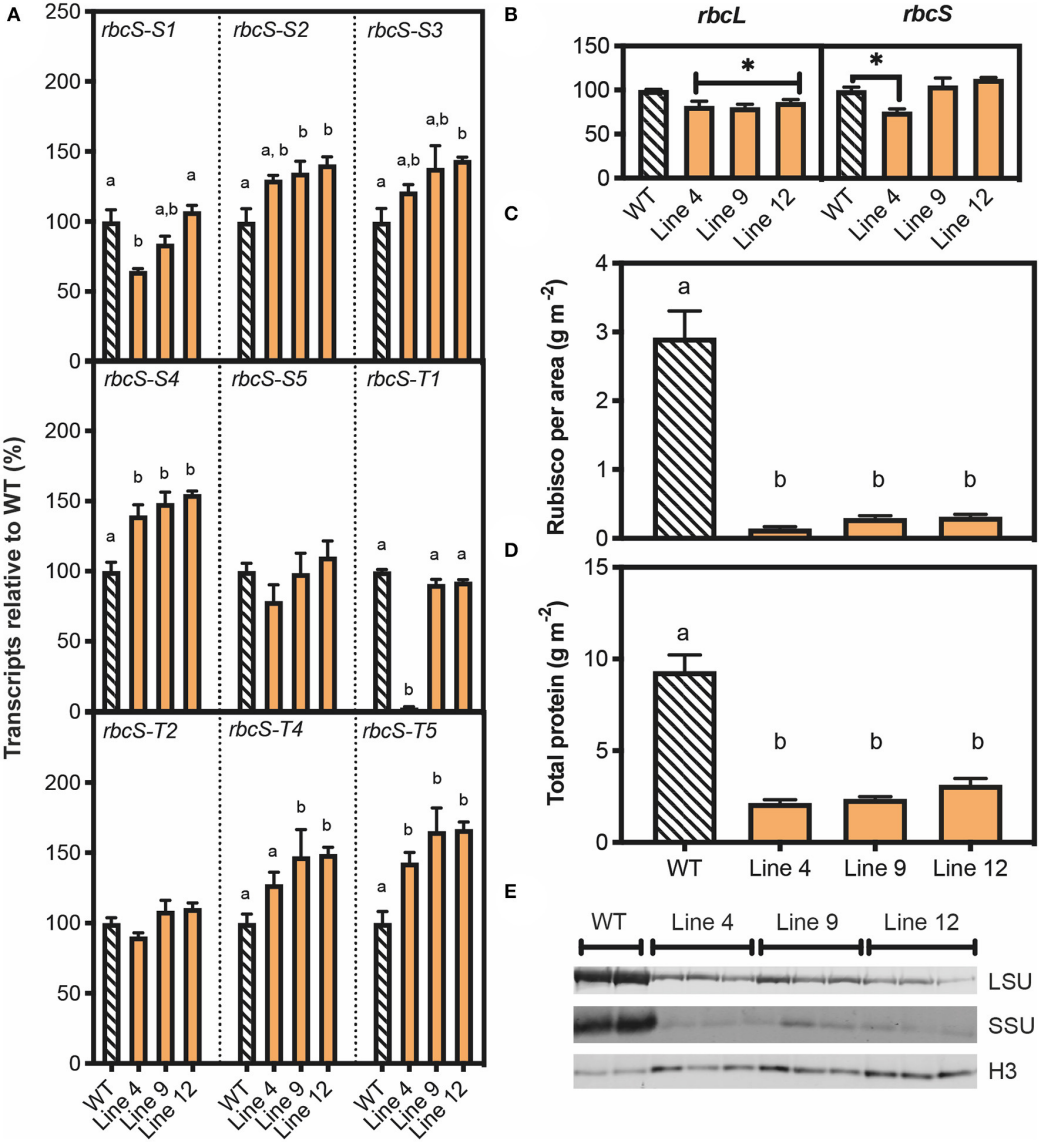
Molecular analyses of T_1_ plants with *SpCas9-*induced mutations in *rbcS-T1*. **(A)** RNA was extracted from 45-day-old plants and the abundance of transcripts for nine *rbcS* homologs was measured by quantitative PCR (qPCR) with gene-specific primers and the transcript level is shown relative to wild-type (Supplementary Information 1 in Supplementary Material). **(B)** Abundance of Rubisco large subunit transcripts (*rbcL)* and total *rbcS* transcripts. The latter was calculated by combining the results for the nine individual *rbcS* homologs. **(C)** Rubisco content per leaf area and **(D)** total protein per leaf area measured by Bradford assay. **(E)** Immunoblot of total soluble protein to detect Rubisco large subunit (LSU) and small subunit (SSU) contents, with a histone H3 (H3) loading control. Protein extracted from an equivalent amount of leaf area was loaded. Each lane represents a biological replicate (two for WT and three for each mutant line). All data are the mean ± SEM of three biological replicates. Significant differences between groups (*P* < 0.05) as identified by one-way ANOVA and Tukey's HSD test are shown by different letters.

Given the pale leaf phenotype of T_1_ plants for lines 4, 9, and 12, and the confirmed mutations in *rbcS-T1*, more in-depth characterisations were carried out on the T_1_ plants for these lines. Initially, this was by determining the relative abundance of Rubisco-encoding transcripts via qRT-PCR (Figures 3A,B). For the three *rbcS* genes targeted for editing by *SpCas9*, transcript levels for *RbcS-T1* and *RbcS-S1a/b* in line 4 were decreased by 98% and 35%, respectively (Figure 3A). In contrast, expression levels were not reduced in lines 9 and 12. Although the relative expression levels of *rbcS-S2, rbcS-S3, rbcS-S4* and *rbcS-T5* were increased in some or all the three mutant lines, this had no significant impact on total relative *rbcS* abundances (Figure 3B). Overall, the total abundance of *rbcS* transcripts for line 4 was also reduced by 25%, while lines 9 and 12 showed no changes in *rbcS* transcript abundance compared to wild-type. All three mutant lines had a 20% reduction in transcripts encoding the large subunit of Rubisco (*rbcL*) relative to wild-type (Figure 3B). Therefore, the reduction in Rubisco content suggested that the three *rbcS* genes had loss-of-function mutations in all three lines.

Remarkably, the leaf Rubisco content in all three mutant lines was decreased by *ca*. 93% relative to wild-type, which corresponded to an 85 and 60% reduction in SSU and LSU, respectively (Figures 3C,E). Total leaf soluble protein content was reduced in lines 4, 9 and 12 by 70–80% (Figure 3D). Our results showed that wild-type plants invested one third of total soluble protein into the Rubisco pool. Thus, the observed decreases in soluble protein for lines 4, 9, and 12 could not be accounted for by the reduction in Rubisco content alone, which indicated that the synthesis of proteins other than Rubisco was also reduced. Furthermore, the mutant lines also had a significant reduction in chlorophyll per leaf area compared to wild-type (Supplementary Figure 5).

### Decreased Rubisco Resulted in Reduced Biomass Accumulation and Lower CO_2_ Assimilation Rates

**Figure 4:**
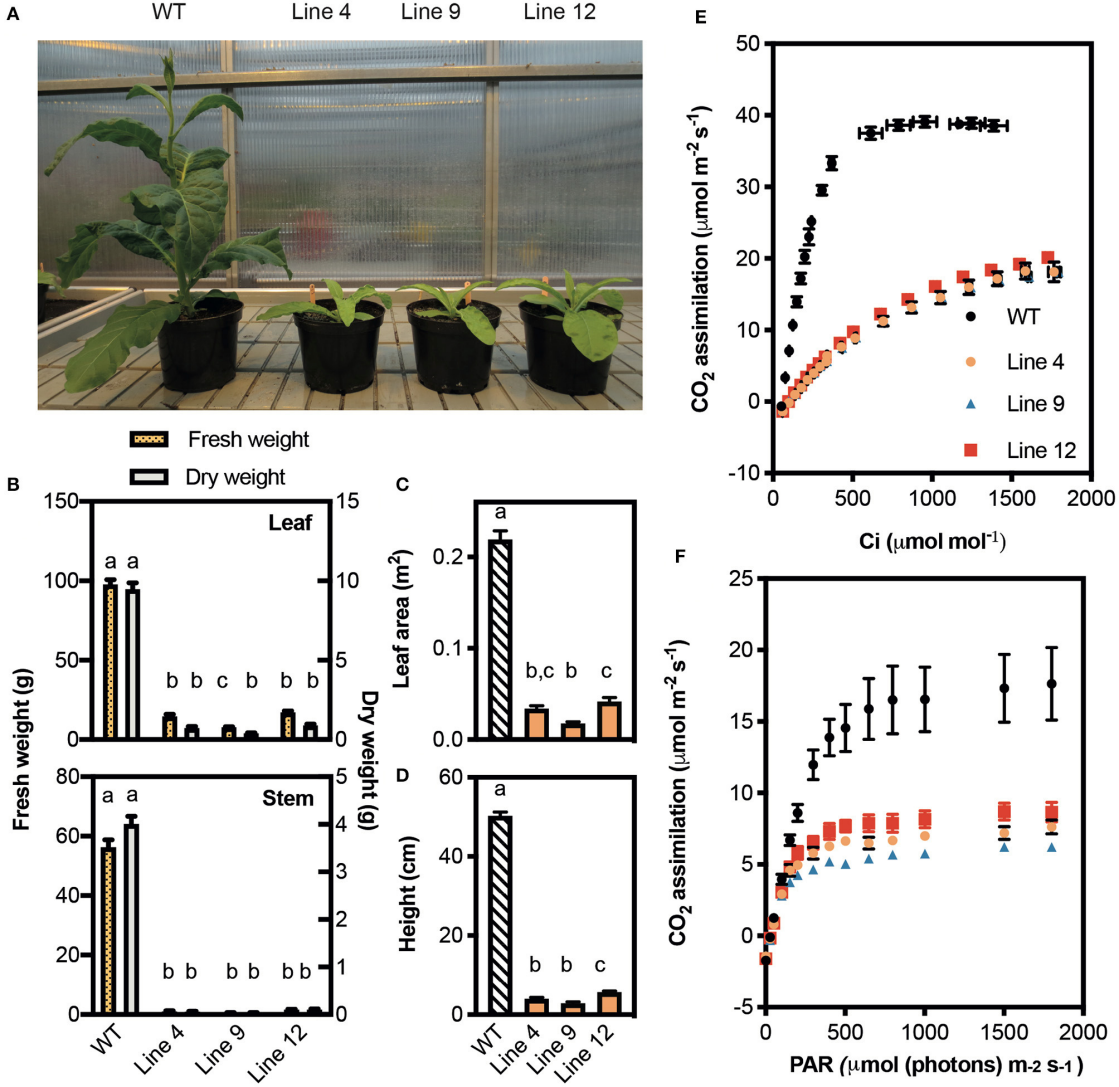
Growth and photosynthetic rates of CRISPR-Cas9 Rubisco mutants in the T_1_ generation. **(A)** Phenotypes of wild-type (WT) and three Rubisco mutant lines after 45 days of growth in a greenhouse with a 15-h photoperiod. **(B)** Fresh (left y-axis) and dry (right y-axis) weights of leaves (top panel) and stems (bottom panel). **(C)** Total leaf area and **(D)** plant height. Growth data show the mean ± SEM (*n* = 6–8) with significant differences (*P* < 0.05) indicated by different letters determined by ANOVA and Tukey's HSD tests. **(E)** The response of *A* to changing irradiance (PAR) measured at 25°C under 400 μmol mol^−1^ CO_2_. **(F)** The response of CO_2_ assimilation (*A*) to intracellular CO_2_ concentration (*C*_i_ ) measured at 25°C under saturating irradiance (1,500 μmol photons m^−2^ s^−1^). Each data point represents the mean ± SEM of four leaves each from separate plants (*n* = 4).

The growth of lines 4, 9, and 12 was compared with that of a non-transformed tissue culture control line (i.e., wild-type plants) (Figures 4A–D). All three mutant lines grew slowly and accumulated <8% of the total biomass (dry weight) of wild-type plants after 45 days of growth. This was associated with a 92% reduction in height, and an 81–93% reduction in total leaf area.

At ambient CO_2_ concentrations (i.e., 400 μmol mol ^−1^) lines 4, 9, and 12 showed similar reductions in the light-saturated rate of CO_2_ assimilation at ambient CO_2_ (*A*_sat_) (*ca*. 42% of wild-type) (Figure 4E, Table 3). CO_2_ assimilation rates also plateaued at a lower light intensity (400 μmol photons m^−2^ s^−1^) compared to wild-type plants (1,000 μmol photons m^−2^ s^−1^). Under saturating light (1,500 μmol photons m^−2^ s^−1^), the response of *A* to changes in *C*_i_ was also affected in the three mutant lines (Figure 4F). The initial slope of the *A*/*C*_i_ response curve is associated with the carboxylation efficiency of Rubisco, and was significantly lower in the mutant lines compared to wild-type (Long and Bernacchi, 2003). Furthermore, the maximum rate of Rubisco carboxylation (*V*_cmax_) was more than 50% lower than wild-type in the mutant lines.

**Table 3:** Photosynthetic parameters of CRISPR-Cas9 plants with reduced rubisco contents.

	**WT**	**Line 4**	**Line 9**	**Line 12**
*A*_sat_ (μmol CO_2_ m^−2^ s^−1^)	21.6 ± 0.8^a^	9.3 ± 0.3^b^	9.4 ± 0.3^b^	9.8 ± 0.4^b^
*V*_cmax_ (μmol CO_2_ m^−2^ s^−1^)	98.9 ± 1.2^a^	41.2 ± 5.4^b^	48.7 ± 2.2^b^	43.8 ± 7.5^b^
Γ (μmol CO_2_ mol^−1^)	53.0 ± 0.5^a^	96.7 ± 1.8^b^	92.1 ± 1.8^b^	95.7 ± 2.9^b^
Initial slope (*A/C*_i_)	0.137 ±0.004^a^	0.027 ± 0.005^b^	0.026 ± 0.004^b^	0.030 ± 0.001^b^
F_v_/*F*_m_	0.85 ± 0.03^a^	0.76 ± 0.02^b^	0.67 ± 0.02^c^	0.77 ± 0.02^b^

*For measurements of net photosynthetic CO_2_ assimilation (A) response to photosynthetically active radiation (PAR) ambient CO_2_ levels were maintained at 400 μmol mol^−1^. Measurements of A in response to sub-stomatal CO_2_ concentration (C_i_) were done under a constant illumination of 1,500 μmol photons m^−2^ s^−1^. All measurements were taken with leaf temperature held at 25°C and under a relative humidity of 60–70%. Values from leaf gas exchange measurements represent the mean ± SEM (n = 4). Dark-adapted leaves were used for F_v_/F_m_ measurements (n = 10). Different letters (i.e. a, b or c) after each value indicate significant differences determined by ANOVA followed by Tukey's HSD tests (P < 0.05). Γ, CO_2_ compensation point; A_sat_, light-saturated CO_2_ assimilation rate at ambient CO_2_; F_v_/F_m_, maximum quantum yield of photosystem II (PSII); V_cmax_, maximum rate of Rubisco carboxylation; WT, wild-type*.

### Co-transformation Facilitated Simultaneous Knockout and Introduction of Rubisco Small Subunits

To test if we could simultaneously reduce native Rubisco content and introduce a novel Rubisco SSU, we co-transformed wild-type tobacco with the plasmid vector pGRNA14 and a second vector carrying an expression cassette for the *CrrbcS2* gene from the green alga Chlamydomonas (pRBCS-Cr) (Supplementary Figure 2). We first designed a suitable expression cassette for the heterologous SSU by testing three common high strength promoters in tobacco protoplasts using a dual-luciferase assay (Supplementary Figure 6A). The *SlrbcS2* gene promoter showed significantly higher activity than the Arabidopsis *rbcS1A* gene promoter, and the Arabidopsis *rbcS3B* gene promoter produced the lowest expression. Therefore, the *SlRbcS2* promoter was chosen to drive *CrrbcS2* expression. The *CrrbcS2* gene was previously modified for expression in higher plants, where the mature peptide was fused to a *rbcS1A* chloroplast transit peptide (Atkinson et al., 2017). Agroinfiltration of tobacco leaves with the modified *CrrbcS2* fused to a GFP-tag at the C-terminus confirmed that the heterologous SSU localized to the chloroplast (Supplementary Figure 6B).

**Figure 5:**
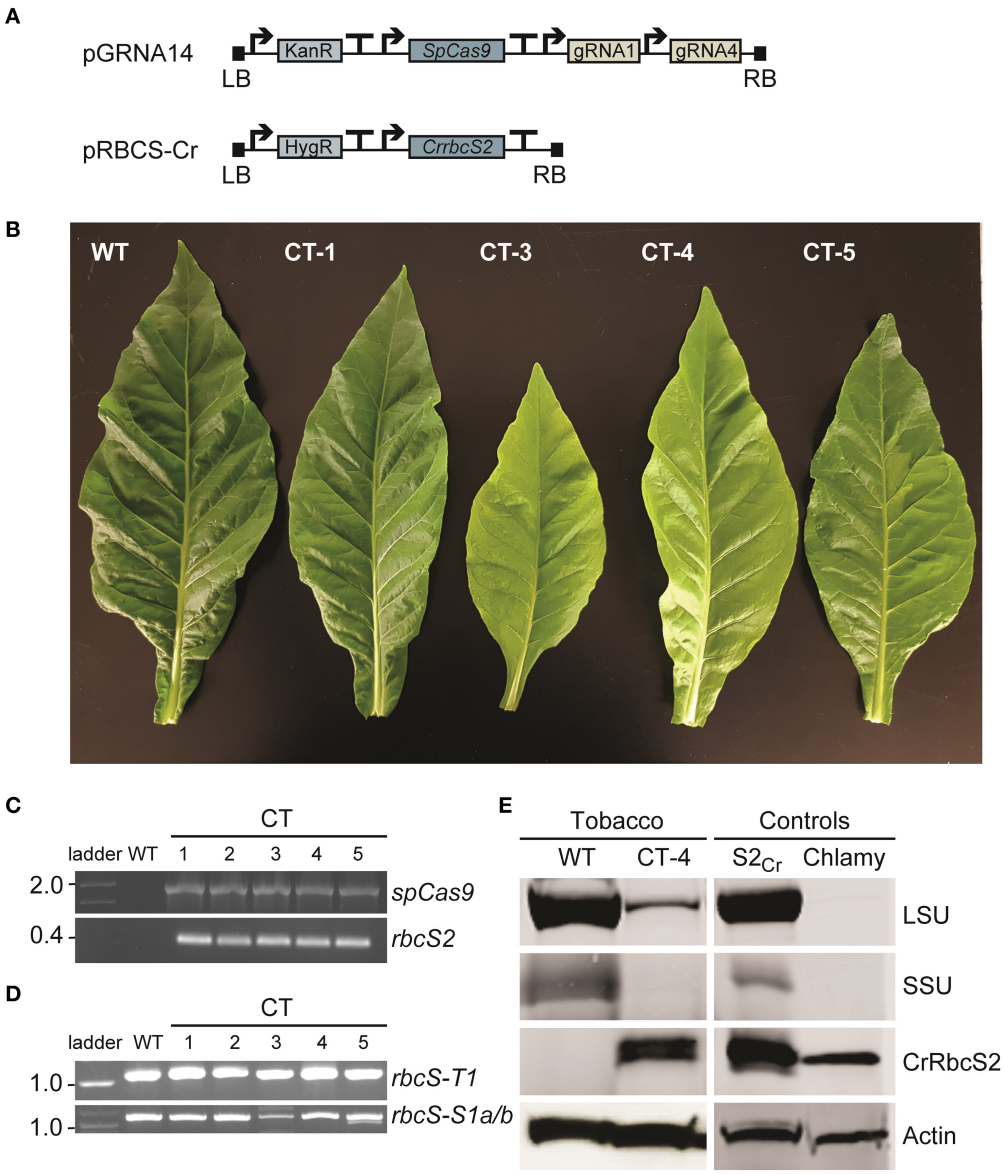
Phenotypes and molecular analyses of co-transformed (CT) plants. **(A)** Wild-type plants were transformed with a Cas-gRNA vector (vector pGRNA14) and gene expression cassette for *CrrbcS2* from Chlamydomonas (vector pRBCS-Cr) (see Supplementary Figures 1, 2 for vector details). **(B)** Leaves of a wild-type (WT) plant and four CT lines with phenotypes ranging from wild-type-like (CT-1) to pale (CT-3 and CT-4) and chimeric (CT-5) are shown. **(C)** PCR amplification of cDNA showing stable integration and expression of *SpCas9* and *rbcS2* in T_0_ CT lines. **(D)** PCR amplification of *rbcS-T1* and *rbcS-S1a/b* (as in Figure 2B). **(E)** Immunoblots of total soluble protein to detect the Rubisco large subunit (LSU) and small subunit (SSU) from tobacco and SSU from Chlamydomonas (CrRbcS2) in CT-4 (T_1_ generation). The two controls were from Arabidopsis expressing CrRbcS2 (S2_Cr_ ; Atkinson et al., 2017) and Chlamydomonas. An actin loading control is shown. No LSU and SSU bands were visible for the Chlamydomonas extract using the primary antibody against wheat Rubisco.

Vectors pGRNA14 and pRBCS-Cr were co-transformed into wild-type tobacco and the explants were cultured on selective media containing two antibiotics (i.e., selective for each T-DNA insertion) (Figures 5A,B). We obtained a small number of plants that had both T-DNA cassettes integrated (*n* = 5) (Figure 5C). Two T_0_ plants (co-transformed (CT) lines CT-3 and CT-4) had a pale leaf phenotype compared to wild-type. Neither of these two lines had a large deletion in *rbcS-T1* (Figure 5D) but sequencing of the *rbcS-T1* amplicon revealed multiple mutated alleles in CT-3 and two mutated alleles in CT-4 (1 bp deletion and 1 bp insertion). Therefore, the CT-3 line was chimeric and the CT-4 line was either chimeric or bi-allelic.

We germinated T_1_ seeds from CT-4 on soil to screen for progeny that contained the *CrrbcS2* transgene but lacked the *SpCas9* transgene. Inheritance of the *CrrbcS2* transgene followed a 3:1 Mendelian segregation (13/20 plants). However, all CT-4 plants screened contained *SpCas9*, indicating multiple copy insertions of the *SpCas9* transgene (Tizaoui and Kchouk, 2012). As a proof-of-principle, we evaluated the mutations in *rbcS-T1* in eleven plants. The wild-type allele was not identified in any of the CT-4 plants that were screened. Four out of eleven plants contained a homozygous 1 bp insertion in *rbcS-T1* (CT-4-i4), while the remaining seven plants had a bi-allelic mutation (9 bp deletion and 1 bp insertion; CT-4-d9) (Supplementary Figure 7A).

Based on a new reference genome for tobacco (Edwards et al., 2017), it was then possible to design primers to differentiate between mutations in *rbcS-S1a* and *rbcS-S1b* (Supplementary Table 2). We re-germinated T_1_ CT-4 seeds and examined four CT-4 plants, in which we identified mutations in all three target *rbcS* genes near the gRNA4 target site, including deletions in *rbcS-S1a* (1-4 bp) and *rbcS-S1b* (2-12 bp), and a bi-allelic 1 bp insertion in *rbcS-T1* (Supplementary Figure 7B). We also re-evaluated tissue samples from each T_0_ line, four T_1_ CRISPR-Cas9 line 4 plants and three T_1_ line 9 plants (see section Transgene-Free Mutants Were Identified in the T1 Generation). In the T_0_ generation, we again identified mutations near the gRNA4 target site, including deletions in *rbcS-S1a* and *rbcS-S1b* (1–4 bp) (Supplementary Figure 7C). Similarly, we found mutations in the T_1_ generation for line 4 and line 9, including deletions in *rbcS-S1a* (1–5 bp) and *rbcS-S1b* (3-12 bp) (Supplementary Figure 7D). Together, these results confirmed that CT-4, line 4 and line 9 had loss-of-function mutations in the T_1_ generation in all three target genes.

Immunoblotting revealed that CrRbcS2 was expressed in T_1_ CT-4 plants (Figure 5E). The size of the band was consistent with that for CrRbcS2 expressed in Arabidopsis [i.e., previously generated line S2_Cr_ (Atkinson et al., 2017)] and Chlamydomonas. Although the T_1_ CT-4 plants expressing CrRbcS2 still contained the *SpCas9* transgene, we performed a preliminary growth analysis to compare this line to three CRISPR-Cas9 SSU lines (i.e., lines 4, 9, and 12) and wild-type plants (Supplementary Figure 8). This analysis showed that the CT-4 line remained smaller that wild-type but accumulated significantly more biomass relative to the CRISPR-Cas9 lines (Supplementary Figures 8A–D). Furthermore, the response of *A* to PAR was significantly higher (*ca*. 45% of wild-type) compared to the CRISPR-Cas9 lines (Supplementary Figures 8E,F). Together, these results indicated that expression of a non-native SSU from Chlamydomonas likely rescued reduction in Rubisco content caused by mutations in three native *rbcS* genes in tobacco. However, further analyses of additional independent CT lines lacking the *SpCas9* transgene are needed to strengthen these findings.

## Discussion

In this study we generated tobacco mutants with decreased amounts of Rubisco by targeting three *rbcS* homologs with CRISPR-Cas9. These lines have a similar decrease in Rubisco content as previous antisense tobacco lines and demonstrate the potential for RGEN-mediated editing of *rbcS* families in polyploid crop species (Khumsupan et al., 2020; Martin-Avila et al., 2020). We also co-transformed tobacco with CRISPR-Cas9 to reduce expression of the native rbcS alongside expression of a non-native rbcS, demonstrating the usefulness of this approach for efforts aimed at improving the efficiency of carbon assimilation through better Rubiscos.

Our strategy was designed to create a 670 bp deletion in three target genes to facilitate simpler screening for multiple mutations by PCR. Transient expression assays in tobacco protoplasts indicated that the dual gRNA approach created large deletions in the target genes with high efficiency. However, *in planta* the large deletion only occurred at a frequency of 12.5% (1/8 plants) in a single target gene (*rbcS-T1*). Our results showed that indel mutations at a single gRNA site were more common than a deletion between both sites in tobacco plants, which is consistent with previous studies in Arabidopsis, *Nicotiana benthamiana, Z. mays*, and *O. sativa* (Zhou et al., 2014; Ordon et al., 2017; Durr et al., 2018; Doll et al., 2019; Khumsupan et al., 2020). Transient assays offer a useful system to pre-select gRNA candidates before stable expression, as differences in the mutation efficiency of each gRNA can reduce the efficiency of deletions between two target sites (Zhou et al., 2014; Doll et al., 2019). However, in agreement with our findings, other studies have reported a lower frequency of large deletions in transgenic plants with gRNAs that appeared highly efficient in transient assays (Zhou et al., 2014; Khumsupan et al., 2020). Therefore, large deletions are feasible but a higher abundance of indels limits the use of paired gRNAs to streamline screening approaches. Reducing the size of the deletion to 50–100 bp could improve the frequency of deletions between two gRNA sites and allow detection by PCR (Ordon et al., 2017).

Few studies have reported RGEN-mediated editing in tobacco and the germline transmission rate of mutations has not yet been described (Gao et al., 2015; Xie et al., 2017). In line with previous studies, we found that most of the *rbcS-T1* alleles in T_0_ plants had mutations at a single gRNA target site. Although homozygous, heterozygous, and bi-allelic mutants have been reported in several species, somatic mutations are more frequently detected (Brooks et al., 2014; Zhang et al., 2014). Non-somatic mutations are likely detected at variable frequencies because of genomic differences in the target site and the timing of DSB repair (Zhang et al., 2014). However, chimeric plants can transmit heritable mutations in germline cells to the next generation (Feng et al., 2014; Zhang et al., 2014). We found complex and variable segregation patterns of mutations in independent lines and identified heritable mutations in the transgene-free progeny of two lines (line 9 and line 12) that were chimeric in the T_0_ generation. Although only a single line (line 4) had the expected 3:1 segregation ratio for *SpCas9*, our results suggest that plants with somatic mutations can be bypassed by screening for mutations in *SpCas9* segregants. As a result, it is possible to increase the likelihood of obtaining transgene-free homozygous mutants in the T_1_ generation in complex polyploid species.

In addition to describing the germline transmission of mutations in tobacco, our work has generated results that offer a useful comparison between RGEN-mediated approaches and antisense technology. Consistent with the reports for antisense *rbcS* tobacco, we found that a severe reduction in Rubisco content reduced photosynthetic rates and biomass accumulation in the CRISPR-Cas9 lines (Quick et al., 1991b; Stitt et al., 1991; Masle et al., 1993; Martin-Avila et al., 2020). Evidently, the antisense tobacco lines had significantly less *rbcS* mRNA than wild-type and Rubisco content was correspondingly decreased. Although our approach produced lines with a more severe decrease in Rubisco content, total *rbcS* transcripts in the CRISPR-Cas9 lines were equivalent to wild-type except for line 4. We hypothesized that the observed reductions in *rbcS* mRNA in line 4 were due to the large deletion, which removed the forward primer binding site and could have disrupted transcription. In contrast, lines 9 and 12 had small indels in *rbcS-T1* that were unlikely to affect transcription and primer binding. The effect of the large deletion on gene transcription could be further investigated in line 4 by designing primers that anneal outside of the deletion region.

The CRISPR-Cas9 lines also had a slight suppression in *rbcL* transcripts that did not seem to be linked to the amount of *rbcS* mRNA. Antisense tobacco lines with *ca*. 12% of wild-type *rbcS* had no observable changes in the amount of *rbcL* mRNA but less LSU protein was produced (Rodermel et al., 1996). In contrast, the CRISPR-Cas9 lines generally had wild-type *rbcS* levels, with the exception of line 4. Therefore, the transcription of *rbcL* in the CRISPR-Cas9 plants is likely affected by different regulatory mechanisms than in the antisense plants because of the lack of, or a relatively small, suppression of *rbcS* mRNA levels (Wostrikoff and Stern, 2007). Alternatively, inhibition of *rbcL* transcription could occur in the CRISPR-Cas9 lines owing to increased degradation of truncated or non-functional SSU peptides, as has been reported for the *polygalacturonase* (PG) gene in tomato (Smith et al., 1990). Similarly, a significant reduction in *rbcL* mRNA was observed in CRISPR-Cas9 and T-DNA insertion Arabidopsis mutants with 3–4% of wild-type *rbcS* (Khumsupan et al., 2020).

Recently, an RNAi-rbcS tobacco master line (tobRrΔS) was described that enables the expression of homogenous non-native Rubisco enzymes by introducing an rbcL-rbcS operon into the plastome of tobacco (Martin-Avila et al., 2020). Our study generated a complementary tobacco line with a stable decrease in Rubisco content to use as a platform for heterologous SSU expression by nuclear transformation. Furthermore, our approach could be extended to crop species that are not amenable to chloroplast transformation. We also co-transformed wild-type tobacco with CRISPR-Cas9 and an heterologous SSU expression vector to examine if it was feasible to simultaneously remove and replace native SSUs, as transformation is time-consuming in non-model and/or crop species (Martin-Avila et al., 2020; Matsumura et al., 2020). Further analyses are required in the next generation of CT-4 plants to determine the amount of Rubisco in these lines and confirm that expression of the Chlamydomonas SSU complemented the reduced growth phenotype produced from simultaneously mutating three *rbcS* genes. However, our finding demonstrate the practicality of a co-transformation approach to circumvent lethal deletions in crops (e.g., when attempting to knockout an entire native *rbcS* family). In conclusion, this proof-of-principle study provides an approach to partially or fully replace entire gene families in a single step in complex polyploid species.

## Data Availability Statement

The raw data supporting the conclusions of this article will be made available by the authors, without undue reservation.

## Author Contributions

AM and SD planned and designed the research and wrote the manuscript. YM and DO performed additional experimental work. All authors assisted with editing the manuscript.

## Conflict of Interest

The authors declare that the research was conducted in the absence of any commercial or financial relationships that could be construed as a potential conflict of interest.
