# Supplementary material for: CRISPR-Cas9-Mediated Mutagenesis of the Rubisco Small Subunit Family in Nicotiana tabacum
Source: Front Genome Ed. 2020 Dec 23;2:605614. doi: 10.3389/fgeed.2020.605614 (PMC8525408; doi:10.3389/fgeed.2020.605614)
Supplement: Supplementary file 1 [file Data_Sheet_1.docx]

**CRISPR-Cas9-mediated mutagenesis of the Rubisco small subunit family in *Nicotiana tabacum***

**Sophie Donovan^1^, Yuwei Mao^1^, Douglas J. Orr^2^, Elizabete Carmo-Silva^2^, Alistair J. McCormick^1^**

^1^SynthSys & Institute of Molecular Plant Sciences, School of Biological Sciences, University of Edinburgh, Edinburgh, EH9 3BF, UK

^2^Lancaster Environment Centre, Lancaster University, Lancaster, LA1 4YQ, UK

**Supplementary Material**

**Supplementary Table 1.** Off-target analysis of gRNA sequences gRNA1-1 and gRNA1-4 targeting three tobacco *rbcS* homologs.

**Supplementary Table 2.** Oligonucleotide sequences for gRNA cloning and PCR screening of tobacco.

**Supplementary Table 3.** Oligonucleotide sequences for quantitative PCR of tobacco *rbcS* genes.

**Supplementary Figure 1.** pGRNA14 vector used for CRISPR-Cas9 editing in tobacco.

**Supplementary Figure 2.** pRBCS-Cr vector used to express a Rubisco small subunit from *Chlamydomonas reinhardtti* in tobacco.

**Supplementary Figure 3.** Alignment of *rbcS-T1, rbcS-S1a*, and *rbcS-S1b* genomic DNA sequences*.*

**Supplementary Figure 4.** Sequence alignment of *rbcS-T1* from the T_0_ CRISPR-Cas9 mutant line 4 and a wild-type plant.

**Supplementary Figure 5.** Chlorophyll content in plants with reduced Rubisco content.

**Supplementary Figure 6.** Transient assays for heterologous expression of a Chlamydomonas Rubisco small subunit in tobacco.

**Supplementary Figure 7.** Sequence analysis of Rubisco small subunit mutants in tobacco produced using CRISPR-Cas9.

**Supplementary Figure 8.** Growth and photosynthetic rates of three tobacco CRISPR-Cas9 Rubisco small subunit mutants and a CRISPR-Cas9 mutant expressing an algal Rubisco small subunit.

**Supplementary Table 1.** Off-target analysis of gRNA sequences gRNA1-1 and gRNA1-4 targeting three tobacco *rbcS* homologs. Results were generated using the Cas-OFFinder tool ([www.rgenome.net/cas-offinder](http://www.rgenome.net/cas-offinder)) and filtered to show off-target sites in the *Nicotiana tabacum* (AYMY01) genome that possessed between 0 and 2 mismatches to the gRNA sequences (Bae et al., 2014). Three sites were identified that contained at least a 1-nt mismatch (bold lowercase) in the 8-12 nt seed region upstream of the PAM motif (underlined).

| gRNA | DNA | Chromosome | Position | Direction | Mismatches |
| --- | --- | --- | --- | --- | --- |
| gRNA1-1 | AAT**c**TTGCTCAA**a**CTAACAGGG | 5337.1 | 173940 | - | 2 |
| gRNA1-1 | AATGTTG**t**TCAAGC**a**AACAAGG | 41530.1 | 39722 | - | 2 |
| gRNA1-4 | AGGCCTGGATC**a**G**a**ATCATTGG | 58211.1 | 1582 | - | 2 |

|  |  |  |  |  |  |
| --- | --- | --- | --- | --- | --- |

| Target | Primer | Sequence | T_a_ (°C) | | Notes / References |
| --- | --- | --- | --- | --- | --- |
| gRNA1 | F | TGTGGTCTCTAGCGAAAAAAAGCACCGACTCGGTGCCAC | - | | Primers for gRNA1 cloning |
|  | R | TGTGGTCA**AATGTTGCTCAAGCTAACA**GTTTAAGAGCTATGCTGGAAACAG | - | |  |
| gRNA4 | F | TGTGGTCTCTAGCGAAAAAAAGCACCGACTCGGTGCCAC | - | | Primers for gRNA4 cloning |
|  | R | TGTGGTCA**AGGCCTGGATCCGTATCAT**GTTTAAGAGCTATGCTGGAAACAG | - | |  |
| spCas9 | F | CGCTAATCTTGCAGGTAGCC | 58 | | Screening primers for SpCas9 integration |
|  | R | AGCCCCGTAATTGACTGATG |  | |  |
| CrrbcS2 | F | GCCGAGAGCGATAAAGCCTA | 60 | | Screening primers for CrrbcS2 integration |
|  | R | CGGCCTCTGTACAAGGAACC |  | |  |
| *rbcS-S1a/b* | F1 | TAGGGTGGTGGGCAACTATG | 64 | |  |
|  | R1 | TTCAAACAAACTGCCCCTAAA |  | |  |
|  | F2 | GCAGCAGTTGCCACCCGCAG | 60 | | Gong et al., 2014 |
|  | R2 | GCAATGAAACTGATGCACTGCACTT |  | |  |
| *rbcS-T1* | F1 | TAGGGTGGTGGGCAACTATG | 64 | |  |
|  | R1 | CGGCACAAGAATGTGAAACA |  | |  |
|  | F2 | GCAGCAGTTGCCACTCGCAC | 60 | | Gong et al., 2014 |
|  | R2 | GCAATGAAACTGATGCACTGCACTT |  | |  |
| *rbcS-S1a* | F | CCACTTCTAAACCATGAATATTAGG | 57 | |  |
|  | R | TTCAAACAAACTGCCCCTAAA | |  |  |
| *rbcS-S1b* | F | GAATATTTTGAAACACAAAAATATAT | 57 | |  |
|  | R | TTCAAACAAACTGCCCCTAAA |  | |  |
|  |  |  |  | |  |

**Supplementary Table 2.** Oligonucleotide sequences for gRNA cloning and PCR screening of tobacco. The gRNA1 and gRNA4 sequences are indicated by bold lettering.

**Supplementary Table 3.** Oligonucleotide sequences for quantitative PCR of tobacco *rbcS* genes. All qPCR primers were used at a T_a_ of 60°C. A serial dilution of cDNA was used to generate standard curves for the *rbcS* gene primers and internal reference gene (*L25)*. The standard curves were used to calculate the amplification efficiency (E) from the gradient (m) of the standard curve as E = 10^(-1/m)^ -1 (R^2^ >0.99).

| Gene | Primer | Sequence | Amplicon (bp) | E (%) (m) | References | |
| --- | --- | --- | --- | --- | --- | --- |
| *rbcS-S1a/b* | F | GATACTATGATGGCAGATACTGGAC | 250 | 104 (-3.24) | | Gong et al., 2014  Schmidt and Delaney, 2010 |
|  | R | TTCAAACAAACTGCCCCTAAA |  |  |  |  |
| *rbcS-S2* | F | TCGAGACTGAGCACGGATT | 305 | 100 (-3.33) | |  |
|  | R | GCCCAAGGAGATTCAAACAA |  |  |  |  |
| *rbcS-S3* | F | TCGAGACTGAGCACGGATT | 297 | 93 (-4.49) | |  |
|  | R | TGCAAACAAACTTTCCCTGA |  |  |  |  |
| *rbcS-S4* | F | TCGAGACTGAGCACGGATT | 293 | 95 (-3.46) | |  |
|  | R | GAAAAACCAAAACAGTTTCTCCA |  |  |  |  |
| *rbcS-S5* | F | TCGAGACTGAGCACGGATT | 312 | 87 (-3.66) | |  |
|  | R | GCGATGAAACTGATGCAC |  |  |  |  |
| *rbcS-T1* | F | CTATGACGGCAGATACTGGAC | 250 | 95 (-3.44) | |  |
|  | R | AAATTAAAACAACACAACCCCTAAA |  |  |  |  |
| *rbcS-T2* | F | TCGAGACTGAGCACGGATT | 245 | 101 (-3.29) | |  |
|  | R | GCAATAAAACTGATGCAC |  |  |  |  |
| *rbcS-T4a/b* | F | TCGAGACTGAGCACGGATT | 339 | 98 (-3.38) | |  |
|  | R | GGGGAAAAACACAAGGAGAA |  |  |  |  |
| *rbcS-T5* | F | TCGAGACTGAGCACGGATT | 246 | 106 (-3.18) | |  |
|  | R | ACTTCCCCCGAAGACATAGG |  |  |  |  |
| *rbcL* | F | TTACAAAGGGCGATGCTACC | 157 | n.d. | |  |
|  | R | CAGGGCTTTGAACCCAAATA |  |  |  |  |
| *L25* | F | CCCCTCACCACAGAGTCTGC | 65 | 105 (-3.20) | |  |
|  | R | AAGGGTGTTGTTGTCCTCAATCTT |  |  |  |  |


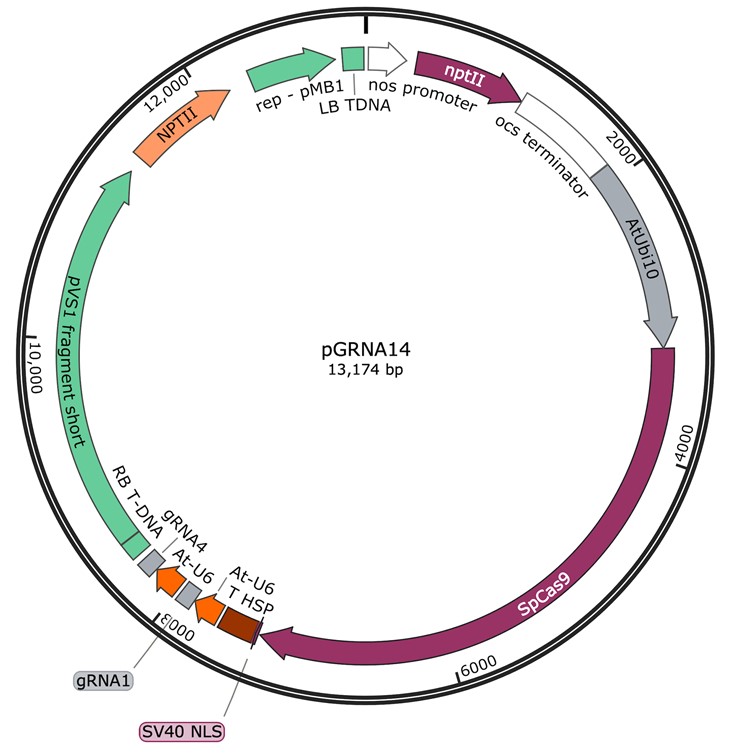


**Supplementary Figure 1.** pGRNA14 vector used for CRISPR-Cas9 editing in tobacco. Vector pGRNA14 was assembled by combining four Level 1 expression cassettes into a Level 2 acceptor vector (pAGM4723). Level 1 position 1 contained the following parts: nos promoter, nopaline synthase promoter (Agrobacterium tumefaciens); nptII, neomycin phosphotransferase II (E. coli); and ocs terminator, octopine synthase terminator (A. tumefaciens) for kanamycin resistance in transformed plants. Level 1 position 2 contained the following parts: AtUbi10, promoter and 5’ UTR, ubiquitin 10 (Arabidopsis thaliana) (pICSL12015); spCas9, cas9 coding sequence (Streptococcus pyogenes) with a nuclear localisation signal (SV40NLS) (pICSL90004); and T HSP, terminator and 3’ UTR, heat shock protein 18.2 (A. thaliana). Level 1 position 3 contained the following parts: At-U6, promoter, U6-26 (A. thaliana) and gRNA1. Level 1 position 4 contained the following parts: At-U6 and gRNA4. The Level 2 acceptor vector contained right border (RB-TDNA) and left border (LB-TDNA) T-DNA sequences, pBM1 and pVS1 origins of replication for E. coli and A. tumefaciens respectively, and neomycin phosphotransferase II (NPTII) resistance for E. coli.


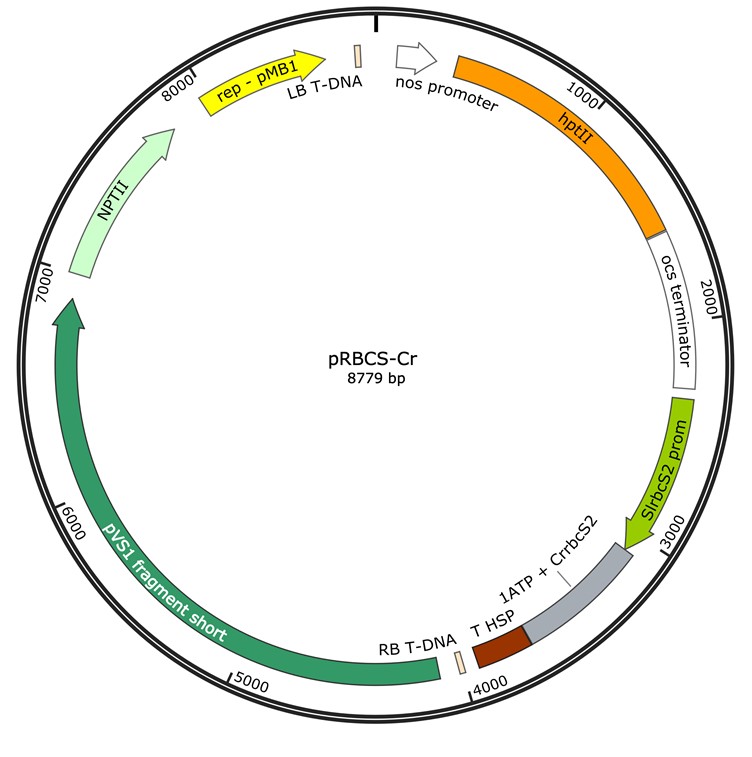


**Supplementary Figure 2.** pRBCS-Cr vector used to express a Rubisco small subunit from Chlamydomonas reinhardtti in tobacco. Vector pRBCS-Cr was assembled by combining two Level 1 expression cassettes into a Level 2 acceptor vector (pAGM4723). Level 1 position 1 contained the following parts: nos promoter, nopaline synthase (A. tumefacians) (pICH87633); hptII, hygromycin phosphotransferase I (pICSL80036); and ocs terminator, octopine synthase (A. tumefaciens) (pICH41432). Level 1 position 2 contained the following parts: SlrbcS2 prom, promoter and 5’ UTR, rbcS2 (Solanum lycopersicum) (pICH71301); 1ATP + CrrbcS2, rbcS1A chloroplast transit peptide (A. thaliana) fused to the mature CrrbcS2 coding sequence (C. reinhardtti); T HSP, terminator and 3’ UTR, heat shock protein 18.2 (A. thaliana). The Level 2 acceptor vector contained right border (RB-TDNA) and left border (LB-TDNA) T-DNA sequences, pBM1 and pVS1 origins of replication for E. coli and A. tumefaciens respectively, and neomycin phosphotransferase II (NPTII) resistance for E. coli.

RbcS-T1 ------------------------------------------------------------ 0

RbcS-S1a CCACTTCTAAACCATGAATATTAGGAAAAACAAGAAA-CAAAACAAATATACATAAACAA 59

RbcS-S1b -TATCAAATTAGTTTGAATATTTTGAAACACAAAAATATATGTATACATACAAAAACAAA 59

RbcS-T1 ------------------------------------------------------------ 0

RbcS-S1a TACGGCTAAAGCCCAAGGAAAAGGGACTCTAAAAAAATTAACCAACCTCAATCACACATT 119

RbcS-S1b TACCGCAATAGTCCAAGCAAAAGGGACTTTAAAAAAAAAAACCAACCTCAACCACACATT 119

RbcS-T1 ------------------------------------------------------------ 0

RbcS-S1a CATATCCTCTTCCTACCCCATCTAGGATGAGATAAGATTACTGAGGTGCTTACACGTGGC 179

RbcS-S1b CATATCCTCTTCCTACCCCATCTAGGATGAGATAAGATTACTGAGGTGCTTACACGTGGC 179

RbcS-T1 ------------------------------------------------------------ 0

RbcS-S1a ACCTCCATTGTGGTGACTA-AATGAAGAGTGGCTTAGCTCAAAATATAATTTTCCAACCT 238

RbcS-S1b ACCTCCATTGTGGTGAATTAAATGATCAATGGCTTAGCTCAAAATATAATTTTCCAACCT 239

RbcS-T1 ------------------------------------------------------------ 0

RbcS-S1a TTCATGTGTGGATATTAAGTTTTGTGTAGTGAATCAAGAACCACATAATCCAATGGTTAG 298

RbcS-S1b TTCATGTGTGGATATTAAGTTTTGTGTAGTGAATCAAGAACCACATAATCCAATGGTTAG 299

RbcS-T1 ------------------------------------------------------------ 0

RbcS-S1a CTTTATTCCAAGATGAGGGGGTTGTTGATTTTTGTCCGTCAGATATAGGAAATATGTAAA 358

RbcS-S1b CTTTACTCCAAGATGAGGGGGTTGTTGATTTTTGTCCGTTAGATATGGGAAATATGTAAA 359

RbcS-T1 ----------------TAGGGTGGTGGGCAACTATGCAATGACCATCTTGGAAGTTAAGG 44

RbcS-S1a ACCTTATCATTATATATAGGGTGGTGGGCAACTATGCAATGACCATATTGGAAGTTAAAG 418

RbcS-S1b ACCTTATCATTATATATAGAGTGGTGGGCAACTATGCAATGACCATCTTGGAAGTTTAAA 419

*** ************************** ********* *

RbcS-T1 AAAAGGGA-------------GAAAGAGAAATCTTTCTGTCTAAAGTGTAATTAGCAATG 91

RbcS-S1a GAAAAGAG------------AGAAAGAGAAATCT-TTCTGTCTAAGTGTAATTAACAATG 465

RbcS-S1b GGAAAAAAAAGGAAAGGGAGAAAGAGAAATCTTT-CTGTCTTAAAGTGTAATTAACAATG 478

** * *** * * * *********** *****

RbcS-T1 GCTTCCTCTGTTCTTTCCTCTGCAGCAGTTGCCACTCGCACC**AATGTTGCTCAAGCTAAC** 151

RbcS-S1a GCTTCCTCAGTTCTTTCCTCTGCAGCAGTTGCCACCCGCAGC**AATGTTGCTCAAGCTAAC** 525

RbcS-S1b GCTTCCTCAGTTCTTTCCTCTGCAGCAGTTGCCACCCGCAGC**AATGTTGCTCAAGCTAAC** 538

******** ************************** **** *******************

RbcS-T1 **A**TGGTTGCACCTTTCACTGGTCTTAAGTCAGCTGCCTCATTCCCTGTTTCAAGGAAGCAA 211

RbcS-S1a **A**TGGTTGCACCTTTCACTGGCCTTAAGTCAGCTGCCTCATTCCCTGTTTCAAGGAAGCAA 585

RbcS-S1b **A**TGGTTGCACCTTTCACTGGCCTTAAGTCAGCTGCCTCATTCCCTGTTTCAAGGAAGCAA 598

******************** ***************************************

RbcS-T1 AACCTTGACATCACTTCCATTGCTAGCAATGGTGGAAGAGTGCAATGCATGCAGGTAACT 271

RbcS-S1a AACCTTGACATCACTTCCATTGCCAGCAACGGCGGAAGAGTGCAATGCATGCAGGTAATT 645

RbcS-S1b AACCTTGACATCACTTCCATTGCCAGCAACGGCGGAAGAGTGCAATGCATGCAGGTAATT 658

*********************** ***** ** ************************* *

RbcS-T1 TATATACATTCGACAATTTTCTTTTTAC------------------AATTATTGTCATAA 313

RbcS-S1a TATATACAATGACAGTGCAAAAAATTTTGATACAATTAATGCATCTTAACATGTCATAGC 705

RbcS-S1b TATATACAATGACAGTGCAAAAAATTTTGATACAATTAATGCATCTTAACATGTCATAGC 718

******** * ** * **

RbcS-T1 TTAAAAGTTGTTTTTGGTGGAGTATAGGTATGGCCCCCATATGGCAAGAAGAAGTACGAA 373

RbcS-S1a TAAAAATTCTATTTTGGTGGAATATAGGTGTGGCCACCAATTAACAAGAAGAAGTACGAG 765

RbcS-S1b TAAAAATTCTATTTTGGTGGAATATAGGTGTGGCCACCAATTAACAAGAAGAAGTACGAG 778

* **** * ********** ******* ***** *** * ***************

RbcS-T1 ACTCTCTCATACCTTCCCGATTTAAGCGAGGAGCAATTGCTTAGTGAAATTGAGTACCTT 433

RbcS-S1a ACTCTCTCATACCTTCCTGATTTGAGCCAGGAGCAATTGCTTAGTGAAGTTGAGTACCTT 825

RbcS-S1b ACTCTCTCATACCTTCCTGATTTGAGCCAGGAGCAATTGCTTAGTGAAGTTGAGTACCTT 838

***************** ***** *** ******************** ***********

RbcS-T1 TTGAAAAATGGATGGGTTCCTTGTTTGGAATTCGAGACTGAGGTCAATAATTTTGCAT-- 491

RbcS-S1a TTGAAAAATGGATGGGTTCCTTGCTTGGAATTCGAGACTGAGGTCAATATCTGTTCTAAA 885

RbcS-S1b TTGAAAAATGGATGGGTTCCTTGCTTGGAATTCGAGACTGAGGTCAATATCTGTTCTAAA 898

*********************** ************************* * * *

RbcS-T1 --------ACTCCCTCTGTTTTATGTGACTTTTTCTTTTTTATATTTGTTGTCCGAGAAA 543

RbcS-S1a TTTTGCATACTCCTTCAATTTTATGCGACATTTTTTTCCTTCTATTTGTTCCAAAAAAAA 945

RbcS-S1b TTTTGCATACTCCTTCAATTTTATGCGACATTTTTTTCCTTCTATTTGTTCCACAAAAAA 958

***** ** ******* *** **** ** ** ******** * ***

RbcS-T1 ACAGA---------------------CATATTTAGAAAAAATTTAACTTTA--------- 573

RbcS-S1a A--AGAAGAAGAAGACGACATATTTATATATTTAGAAAAAATTTAACTTTTAACTTTAAT 1003

RbcS-S1b AATGAAAGAAGAAGACGATATATTTATATATTTAGAAAAAATTTAACTTTTAACTTTAAT 1018

* ***********************

RbcS-T1 AAATTGCTTAATATGTGTAGCGCGGATTTGTCTACCGTGAAAACAACAAGTCACCAGGAT 633

RbcS-S1a ATGTTATTTTGCATGTGCAGCACGGATTTGTCTACCGTGAAAACAACAAGTCACCAGGAT 1063

RbcS-S1b ATATTATTTTGCATGTGCAGCACGGATTTGTCTATCGTGAAAACAACAAGTCACCAGGAT 1078

* ** ** ***** *** ************ *************************

RbcS-T1 ACTATGACGGCAGGTGAGTCACAATTATTTAGT--------TAAATCATGAATAT----- 680

RbcS-S1a ACTATGATGGCAGGTCAGTACCAAATCCACAATATTTTAGTTAAATCATAGATATATGTA 1123

RbcS-S1b ACTATGATGGCAGGTCAGTACCAAATCCACAATATTTTAGTTAAATTATAGATATATGTA 1138

******* ******* *** *** * * * ***** ** ****

RbcS-T1 ----------------------------------------------------------AA 682

RbcS-S1a CGCAGATCTTAAAAAGAAGTACAAAAATCATGTTTCGAGACCATGTTTTAGAAGAACGAA 1183

RbcS-S1b CGCAGATCTTGAAAAGAAGTACA-AAATCATGTTTCGAGACCATGTTTTAGAAGAACGAA 1197

**

RbcS-T1 ATGTTAAAT---------TTTAAATCTTGAATGCGCAGATACTGGACCATGTGGAAGCTG 733

RbcS-S1a ATCTTGAATTTTAAATCTTGAATCTGGTACTGATGCAGATACTGGACCATGTGGAAGCTA 1243

RbcS-S1b ATCTTGAATTTTAAATCCTGAATCCGGTACTGATGCAGATACTGGACCATGTGGAAGCTA 1257

** ** *** * * * *************************

RbcS-T1 CCCATGTTTGGGTGCACTGATGCCACCCAAGTGTTAGCTGAGGTGGGAGAGGCGAAGAAG 793

RbcS-S1a CCTATGTTCGGATGCACTGATGCCACCCAAGTGTTGGCTGAGGTGGAAGAGGCGAAGAAG 1303

RbcS-S1b CCTATGTTCGGATGCACTGATGCCACCCAAGTGTTGGCTGAGGTAGAAGAGGCGAAGAAG 1317

** ***** ** *********************** ******** * *************

RbcS-T1 GCATACCCAG**AGGCCTGGATCCGTATCAT**TGGATTCGACAACGTGCGTCAAGTGCAGTGC 853

RbcS-S1a GCATACCCAC**AGGCCTGGATCCGTATCAT**TGGATTCGACAACGTGCGTCAAGTGCAGTGC 1363

RbcS-S1b GCATACCCAC**AGGCCTGGATCCGTATCAT**TGGATTCGACAACGTGCGTCAAGTGCAGTGC 1377

********* **************************************************

RbcS-T1 ATCAGTTTCATTGCCTACAAGCCTGAAGGCTACTAAGTTACATATTAGGACAACTTCCCT 913

RbcS-S1a ATCAGTTTCATTGCCTACAAGCCAGAAGGCTACTAAGTTTCATATTAGGACAACTTACCC 1423

RbcS-S1b ATCAGTTTCATCGCCTACAAGCCAGAAGGCTACTAAGTTTCATATTAGGACAACTTACCC 1437

*********** *********** *************** **************** **

RbcS-T1 ATTGTCTTGTCTTTAGGGGTTGTGTTGTTTTAATTTTTTTTTACTTCTTCCCACAAAAAC 973

RbcS-S1a TATTGTCTGACTTTAGGGGCAGTTTGTTTGAA---------------------------- 1455

RbcS-S1b TATTGTCTGACTTTAGGGGCAGTTTGTTTGAA---------------------------- 1469

* ** ********* ** * ** *

RbcS-T1 TGTTTATGTTTCCTTCTTTCTATTCGGTGTATGTTTTTCGATTCCTACCAAGTTATGAGA 1033

RbcS-S1a ------------------------------------------------------------ 1455

RbcS-S1b ------------------------------------------------------------ 1469

RbcS-T1 CCTAATAATTATGATTTGGTGCTTTGTTTGTATAATATTTTTGTTTCACATTCTTGTGCC 1093

RbcS-S1a ------------------------------------------------------------ 1455

RbcS-S1b ------------------------------------------------------------ 1469

RbcS-T1 G 1094

RbcS-S1a - 1455

RbcS-S1b - 1469

**Supplementary Figure 3.** Alignment of *rbcS-T1, rbcS-S1a,* and *rbcS-S1b* genomic DNA sequences showing primers and gRNA target sites. Sequences show gene-specific primers for *rbcS-S1a* and *rbcS-S1b* (curved underline), primers rbcS-T1 F1, *rbcS-S1a/b* F1 and R1 (bold underline), gRNA target sites 1 and 4 (bold red).

rbcS-T1_WT 1 T---AGGGTGG-TGGGCAACTATGCAATGACCATCTTGGAAGTTAAGGAA

| ||||||| ||||||||||||||||||||||||||||||||||||||

Line 4 1 TTTTAGGGTGGGTGGGCAACTATGCAATGACCATCTTGGAAGTTAAGGAA

rbcS-T1_WT 47 AAGGGAGAAAGAGAAATCTTTCTGTCTAAAGTGTAATTAGCAATGGCTTC

||||||||||||||||||||||||||||||||||||||||||||||||||

Line 4 51 AAGGGAGAAAGAGAAATCTTTCTGTCTAAAGTGTAATTAGCAATGGCTTC

rbcS-T1_WT 97 CTCTGTTCTTTCCTCTGCAGCAGTTGCCACTCGCACC**AATGTTGCTCAAG**

||||||||||||||||||||||||||||||||||||||||||||||||||

Line 4 101 CTCTGTTCTTTCCTCTGCAGCAGTTGCCACTCGCACCAATGTTGCTCAAG

rbcS-T1_WT 147 **CTAACA**TGGTTGCACCTTTCACTGGTCTTAAGTCAGCTGCCTCATTCCCT

||

Line 4 151 CT------------------------------------------------

rbcS-T1_WT 197 GTTTCAAGGAAGCAAAACCTTGACATCACTTCCATTGCTAGCAATGGTGG

Line 4 153 --------------------------------------------------

rbcS-T1_WT 247 AAGAGTGCAATGCATGCAGGTAACTTATATACATTCGACAATTTTCTTTT

Line 4 153 --------------------------------------------------

rbcS-T1_WT 297 TACAATTATTGTCATAATTAAAAGTTGTTTTTGGTGGAGTATAGGTATGG

Line 4 153 --------------------------------------------------

rbcS-T1_WT 347 CCCCCATATGGCAAGAAGAAGTACGAAACTCTCTCATACCTTCCCGATTT

Line 4 153 --------------------------------------------------

rbcS-T1_WT 397 AAGCGAGGAGCAATTGCTTAGTGAAATTGAGTACCTTTTGAAAAATGGAT

Line 4 53 --------------------------------------------------

rbcS-T1_WT 447 GGGTTCCTTGTTTGGAATTCGAGACTGAGGTCAATAATTTTGCATACTCC

Line 4 153 --------------------------------------------------

rbcS-T1_WT 497 CTCTGTTTTATGTGACTTTTTCTTTTTTATATTTGTTGTCCGAGAAAACA

Line 4 153 --------------------------------------------------

rbcS-T1_WT 547 GACATATTTAGAAAAAATTTAACTTTAAAATTGCTTAATATGTGTAGCGC

Line 4 153 --------------------------------------------------

rbcS-T1_WT 597 GGATTTGTCTACCGTGAAAACAACAAGTCACCAGGATACTATGACGGCAG

Line 4 153 --------------------------------------------------

rbcS-T1_WT 647 GTGAGTCACAATTATTTAGTTAAATCATGAATATAAATGTTAAATTTTAA

Line 4 153 --------------------------------------------------

rbcS-T1_WT 697 ATCTTGAATGCGCAGATACTGGACCATGTGGAAGCTGCCCATGTTTGGGT

Line 4 153 --------------------------------------------------

rbcS-T1_WT 747 GCACTGATGCCACCCAAGTGTTAGCTGAGGTGGGAGAGGCGAAGAAGGCA

Line 4 153 --------------------------------------------------

rbcS-T1_WT 797 TACCCAG**AGGCCTGGATCCGTATCAT**TGGATTCGACAACGTGCGTCAAGT

||||||||||||||||||||||||||||

Line 4 153 ----------------------TCATTGGATTCGACAACGTGCGTCAAGT

rbcS-T1_WT 847 GCAGTGCATCAGTTTCATTGCCTACAAGCCTGAAGGCTACTAAGTTACAT

||||||||||||||||||||||||||||||||||||||||||||||||||

Line 4 181 GCAGTGCATCAGTTTCATTGCCTACAAGCCTGAAGGCTACTAAGTTACAT

rbcS-T1_WT 897 ATTAGGACAACTT-CCCTATTGTCTTGTCTTTAGGGGTTGTGTTGTTTTA

||||||||||||| ||||||||||||||||||||||||||||||||||||

Line 4 231 ATTAGGACAACTTACCCTATTGTCTTGTCTTTAGGGGTTGTGTTGTTTTA

rbcS-T1_WT 946 ATTTTTTTTTACTTCTTCCCACAAAAACTGTTTATGTTTCCTTCTTTCTA

||||||||||||||||||||||||||||||||||||||||||||||||||

Line 4 281 ATTTTTTTTTACTTCTTCCCACAAAAACTGTTTATGTTTCCTTCTTTCTA

rbcS-T1_WT 996 TTCGGTGTATGTTTTTCGATTCCTACCAAGTTATGAGACCTAATAATTAT

||||||||||||||||||||||||||||||||||||||||||||||||||

Line 4 331 TTCGGTGTATGTTTTTCGATTCCTACCAAGTTATGAGACCTAATAATTAT

rbcS-T1_WT 1046 GATTTGGTGCTTTGTTTGTATAATATTTTTGTTTCACATTCTTGTGCCG

||||||||||.|.||| |.||.

Line 4 381 GATTTGGTGCGTGGTT-GAATT---------------------------

**Supplementary Figure 4.** Sequence alignment of *rbcS-T1* from the T_0_ CRISPR-Cas9 mutant line 4 and a wild-type plant. Sequences were obtained from Sanger sequencing of PCR products amplified using gene-specific primers rbcS-T1 F2 and rbcS-T1 R2 (**Supplementary Table 2**). A 670 bp deletion was found in line 4 between the gRNA1 and gRNA4 target sites (bold red).

**Supplementary Figure 5.** Chlorophyll content in CRISPR-Cas9-edited plants with reduced Rubisco content. Chlorophyll a and chlorophyll b content was determined in equivalent leaf area in wild-type plants and mutant lines 4, 9 and 12. Values represent the mean ± SEM (n = 4) followed by different letters indicating significant differences determined by ANOVA followed by Tukey’s HSD tests (P < 0.05).

**Supplementary Figure 6.** Transient assays for heterologous expression of a Chlamydomonas Rubisco small subunit in tobacco. **(A)** A dual-luciferase assay was used to test promoter strength in tobacco mesophyll protoplasts. Protoplasts were transfected with a vector containing the *A. tumefaciens* *nopaline synthase* promoter control (Nos), Arabidopsis *rbcS3B* promoter (*AtrbcS3B*), *rbcS1A* promoter (*rbcS1A*), or *Solanum lycopersicum rbcS2* promoter (*SlrbcS2*) driving a nano luciferase reporter (N_LUC_) and an internal firefly luciferase reporter (F_LUC_). Luciferase activity was measured after exposing protoplasts to 20 µmol photons m^-2^ s^-1^ or 100 µmol photons m^-2^ s^-1^ of light for one hour. The luminescence of N_LUC_ was normalised to the F_LUC_ internal control. Values show the mean ± SEM (n = 4) with different letters showing significant differences determined by ANOVA and Tukey’s HSD (P < 0.05). **(B)** Transient expression in tobacco following agroinfiltration of a modified Chlamydomonas CrRbcS2 (Atkinson et al., 2017) fused to a GFP reporter at the C-terminus. The *CrrbcS2* expression cassette was driven by the *SlrbcS2* promoter with the *AtrbcS1A* transit peptide shows localisation of GFP-tagged SSU to the chloroplast. Green and red signals are GFP fluorescence and chlorophyll autofluorescence, respectively. The overlay image of these signals is shown: overlaps are yellow.

**(A)**

**CT-4 (T_1_)**

*rbcS-T1*

WT AGCTGAGGTGGGAGAGGCGAAGAAGGCATACCCAG**AGGCCTGGATCCGTATCAT**TGGATTCGACAA

CT-4-i4 AGCTGAGGTGGGAGAGGCGAAGAAGGCATACCCAG**AGGCCTGGATCCGTAGTCAT**TGGATTCGACAA +1

CT-4-d9 a1 AGCTGAGGTGGGAGAGGCGAAGAAGGCATACCCAG**AGGCCTGGATCCGTAGTCAT**TGGATTCGACAA +1

CT-4-d9 a2 AGCTGAGGTGGGAGAGGCGAAGAAGGCATACCCAG**AGGCC---------ATCAT**TGGATTCGACAA -9

**(B)**

**CT-4 (T_1_)**

*rbcS-S1a*

WT GGCTGAGGTGGAAGAGGCGAAGAAGGCATACCCAC**AGGCCTGGATCCGTATCAT**TGGATTCGACAA

CT-4-1 GGCTGAGGTGGAAGAGGCGAAGAAGGCATACCCAC**AGGCCTGGATCCGTAT**-**AT**TGGATTCGACAA -1

CT-4-2 GGCTGAGGTGGAAGAGGCGAAGAAGGCATACCCAC**AGGCCTGGATCCGTAT**-**AT**TGGATTCGACAA -1

CT-4-3 GGCTGAGGTGGAAGAGGCGAAGAAGGCATACCCAC**AGGCCTGGATCCGTAT**-**AT**TGGATTCGACAA -1

CT-4-4 GGCTGAGGTGGAAGAGGCGAAGAAGGCATACCCAC**AGGCCTGGATCC**----**CAT**TGGATTCGACAA -4

*rbcS-S1b*

WT GGCTGAGGTAGAAGAGGCGAAGAAGGCATACCCAC**AGGCCTGGATCCGTATCAT**TGGATTCGACAA

CT-4-1 GGCTGAGGTAGAAGAGGCGAAGAAGGCATACCCAC**CGG------------TCTT**TGGATTCGACAA -12, S

CT-4-2 GGCTGAGGTAGAAGAGGCGAAGAAGGCATACCCAC**AGGCCTGGATCCGTAT--T**TGGATTCGACAA -2

CT-4-3 GGCTGAGGTAGAAGAGG**A**GA**C**GAAGGCATACCCAC**AGACC------------AT**TGGATTCGACAA -12, S

CT-4-4 GGCTGAGGTAGAAGAGGCGAAGAAGGCATACCCAC**AGACC------------AT**TGGATTCGACAA -12, S

*rbcS-T1*

WT AGCTGAGGTGGGAGAGGCGAAGAAGGCATACCCAG**AGGCCTGGATCCGTATCAT**TGGATTCGACAA

CT-4-1 a1 AGCTGAGGTGGGAGAGGCGAAGAAGGCATACCCAG**AGGCCTGGATCCGTATACAT**TGGATTCGACAA +1

CT-4-1 a2 AGCTGAGGTGGGAGAGGCGAAGAAGGCATACCCAG**AGGCCTGGATCCGTATTCAT**TGGATTCGACAA +1

CT-4-2 a1 AGCTGAGGTGGGAGAGGCGAAGAAGGCATACCCAG**AGGCCTGGATCCGCATACAT**TGGATTCGACAA +1, S

CT-4-2 a2 AGCTGAGGTGGGAGAGGCGAAGAAGGCATACCCAG**AGGCCTGGATCCGCATTCAT**TGGATTCGACAA +1, S

CT-4-3 a1 AGCTGAGGTGGGAGAGGCGAAGAAGGCATACCCAG**AGGCCTGGATCCGCATACAT**TGGATTCGACAA +1, S

CT-4-3 a2 AGCTGAGGTGGGAGAGGCGAAGAAGGCATACCCAG**AGGCCTGGATCCGCATTCAT**TGGATTCGACAA +1, S

CT-4-4 a1 AGCTGAGGTGGGAGAGGCGAAGAAGGCATACCCAG**AGGCCTGGATCCGCATACAT**TGGATTCGACAA +1, S

CT-4-4 a2 AGCTGAGGTGGGAGAGGCGAAGAAGGCATACCCAG**AGGCCTGGATCCGCATTCAT**TGGATTCGACAA +1, S

**(C)**

**T_0_ lines**

*rbcS-S1a*

WT GGCTGAGGTGGAAGAGGCGAAGAAGGCATACCCAC**AGGCCTGGATCCGTATCAT**TGGATTCGACAA

Line 2 GGCTGAGGTGGAAGAGGCGAAGAAGGCATACCCAC**CCGCCCGGGTCCCT**-**TCAT**TGGATTCGACAA -1, S

Line 3 GGCTGAGGTGGAAGAGGCGAAGAAGGCATACCCAC**ACGCCCGGGTCCCAGTCAT**TGGATTCGACAA S

Line 4 GGCTGAGGTGGAAGAGGCGAAGAAGGCATACCCAC**AGGCCTGGATCC**----**CAT**TGGATTCGACAA -4

Line 9 GGCTGAGGTGGAAGAGGCGAAGAAGGCATACCCAC**CGGCCCGGCTCCGTATCAT**TGGATTCGACAA S

Line 12 GGCTGAGGTGGAAGAGGCGAAGAAGGCATACCCAC**ACGCCCGGGCCGGTATCAT**TGGATTCGACAA S

Line 21 GGCTGAGGTGGAAGAGGCGAAGAAGGCATACCCAC**AGGCCTGGATCCGTATCAT**TGGATTCGACAA

*rbcS-S1b*

WT GGCTGAGGTAGAAGAGGCGAAGAAGGCATACCCAC**AGGCCTGGATCCGTATCAT**TGGATTCGACAA

Line 2 GGCTGAGGTAGAAGAGGCGAAGAAGGCATACCCAC**GGCCCGGGA**-**CCG**--**TCAT**TGGATTCGACAA -3, S

Line 3 GGCTGAGGTAGAAGAGGCGAAGAAGGCATACCCAC**AGGCCTGGCTCCGTATCAT**TGGATTCGACAA S

Line 4 GGCTGAGGTAGAAGAGGCGAAGAAGGCATACCCAC**AGG**-**CTGCAATCAGT**-**TCA**TTGGATTCGACAA -2, +1, S

Line 9 GGCTGAGGTAGAAGAGGCGAAGAAGGCATACCCAC**AGGCCTGGATC----TCAT**TGGATTCGACAA -4

Line 12 GGCTGAGGTAGAAGAGGCGAAGAAGGCATACCCAC**CGGC**---**GAGCC**---**TCAT**TGGATTCGACAA -6, S

Line 21 GGCTGAGGTAGAAGAGGCGAAGAAGGCATACCCAC**AGGCCTGGATCCGTATCAT**TGGATTCGACAA

**(D)**

**Line 4 (T_1_)**

*rbcS-S1a*

WT GGCTGAGGTGGAAGAGGCGAAGAAGGCATACCCAC**AGGCCTGGATCCGTATCAT**TGGATTCGACAA

SSU4-1 GGCTGAGGTGGAAGAGGCGAAGAAGGCATACCCAC**AGGCCTGGATCC**----**CAT**TGGATTCGACAA -4

SSU4-2 GGCTGAGGTGGAAGAGGCGAAGAAGGCATACCCAC**AGGCCTGGATCC**----**CAT**TGGATTCGACAA -4

SSU4-3 GGCTGAGGTGGAAGAGGCGAAGAAGGCATACCCAC**AGGCCTGGATCC**----**CAT**TGGATTCGACAA -4

SSU4-4 GGCTGAGGTGGAAGAGGCGAAGAAGGCATACCCAC**AGGCCTGGATCCGTAT**-**AT**TGGATTCGACAA -1

*rbcS-S1b*

WT GGCTGAGGTAGAAGAGGCGAAGAAGGCATACCCAC**AGGCCTGGATCCGTATCAT**TGGATTCGACAA

SSU4-1 GGCTGAGGTAGAAGAGGCGAAGAAGGC**C**TACCCAC**AG------------ACCAT**TGGATTCGACAA -12, S

SSU4-2 GGCTGAGGTAGAAGAGGCGAAGAAGGCATACCCAC**AG------------ACCAT**TGGATTCGACAA -12, S

SSU4-3 GGCTGAGGTAGAAGAGGCGAAGAAGGCATACCCAC**AGGCC------CGGGTCAT**TGGATTCGACAA -6, S

SSU4-4 GG-----------GAGGCGAAGAAGGCATACCCAC**AGGCCTGGATCCGTATCAT**TGGATTCGACAA -11

**Line 9 (T_1_)**

*rbcS-S1a*

WT GAGGTGGAAGAGGCGAAGAAGGCATACCCAC**AGGCCTGGATCCGTATCAT**TGGATTCGACAA

SSU9-1 GAGGTGGAAGAGGCGAAGAAGGCATACCCAC**AGGCCTGGATCCGTAT**-**AT**TGGATTCGACAA -4

SSU9-2 GAGGTGGAAGAGGCGAAGAAGGCATACCCAC**AGGCATCGATCC**-----**AT**TGGATTCGACAA -4

SSU9-3 GAGGTGGAAGAGGCGAAGAAGGCATACCCAC**ACACAGGCATCGATCC**-----**AT**TGGATTCGACAA +4, -5, S

*rbcS-S1b*

WT GGCTGAGGTAGAAGAGGCGAAGAAGGCATACCCAC**AGGCCTGGATCCGTATCAT**TGGATTCGACAA

SSU9-1 GGCTGAGGTAGAAGAGGCGAAGAAGGCATACCCAC**AGGCCTGGATC**----**TCAT**TGGATTCGACAA -4

SSU9-2 GGCTGAGGTAGAAGAGGCGAAGAAGGCATACCCAC**CGGCCTGGATCC**---**TCAT**TGGATTCGACAA -3, S

SSU9-3 GGCTGAGGTAGAAGAGGCGAAGAAGGCATACCCAC**CGGCCCGGATCC**---**TCAT**TGGATTCGACAA -3, S

**Supplementary Figure 7.** Sequence analysis of Rubisco small subunit mutants in tobacco produced using CRISPR-Cas9. **(A)** Mutations in *rbcS-T1* were confirmed by Sanger sequencing plants in the T_1_ generation of the co-transformed line CT-4. **(B)** Mutations in *rbcS-T1*, *rbcS-S1a* and *rbcS-S1b* were subsequently identified in T_1_ CT-4 progeny. **(C)** Examples of mutations in *rbcS-S1a* and *rbcS-S1b* in the T_0_ generation for the CRISPR-Cas9 lines 2, 3, 4, 9, 12 (no mutations were found in the sample for line 21). **(D)** Mutations in the T_1_ generation for CRISPR-Cas9 lines 4 and 9. The PAM sequences are underlined, gRNA sequences are in bold. Bold red denotes mutations (“+” is insertion, “-” is deletion and “S” is substitution). Bi-allelic mutations are shown with ‘a1’ and ‘a2’. Primer sequences are given in **Supplementary Table 2**.

**
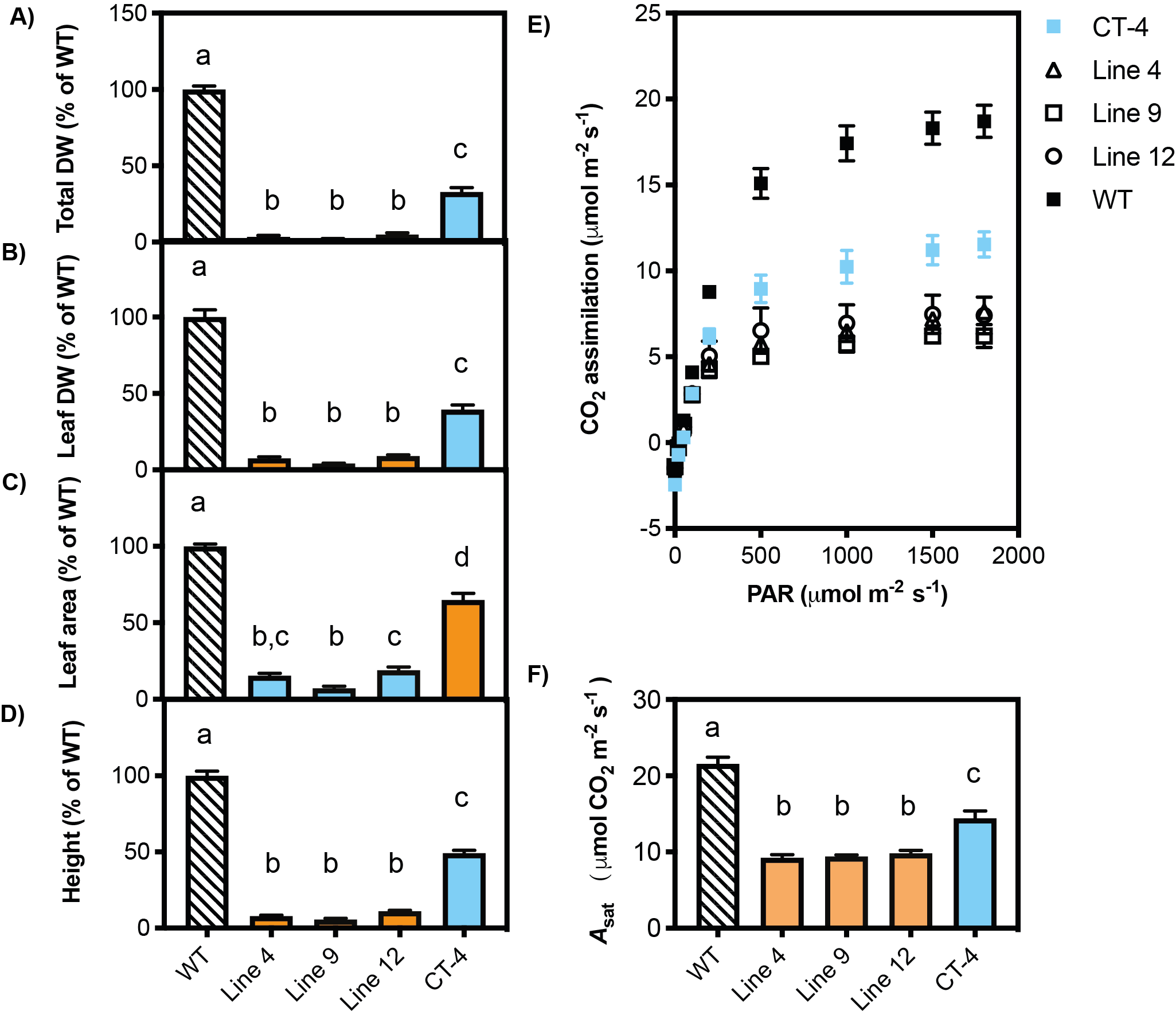
**

**Supplementary Figure 8.** Growth and photosynthetic rates of three tobacco CRISPR-Cas9 Rubisco small subunit mutants and a CRISPR-Cas9 mutant expressing an algal Rubisco small subunit. **(A)** Total dry weight, **(B)** leaf dry weight, **(C)** total leaf area, and **(D)** height of 45-day-old T_1_ tobacco lines with decreased SSU content and a line co-complemented with an SSU from Chlamydomonas (CT-4). Growth data for the three Rubisco mutant lines (n = 6-8) and line CT-4 (n = 10) were collected in two independent experiments and are shown relative to wild-type (n = 10). **(E)** The response of CO_2_ assimilation (*A*) to changing irradiance (PAR) measured at 25 °C under atmospheric CO_2_ concentrations (400 μmol mol^-1^). **(F)** The *A/*PAR response curves were used to derive the light- saturated rate of photosynthesis at ambient CO_2_ (*A*_sat_). Each data point represents the mean ± SEM of four leaves from a separate plant (n = 4). Different letters indicate significant differences determined by ANOVA followed by Tukey’s HSD tests (P < 0.05).
